# CD39 Contributes to the Ability of Cell Invasion in Heterogeneity of Colorectal Cancer

**DOI:** 10.1111/jcmm.70486

**Published:** 2025-03-07

**Authors:** Xiaosong Li, Yifen Shen, Jianhua Lang, Jianzhong Wu, Zhenhai Qian, Genhai Shen, Yihang Shen

**Affiliations:** ^1^ Department of General Surgery Suzhou Ninth People's Hospital Suzhou Jiangsu China; ^2^ Central Laboratory, Suzhou Bay Clinical College Suzhou Ninth People's Hospital, Xuzhou Medical University Suzhou Jiangsu China

**Keywords:** CD39, cell invasion, colorectal cancer cells, SMAD3

## Abstract

Tumour heterogeneity will accumulate and amplify during cell culture and passage, even if derived from the same strain. In the current study, multiple batches of colorectal cancer HCT116 and HT29 cell lines were obtained using different conditions of trypsin digestion and processed for RNA sequencing. CD39 was identified as a biomarker highly expressed in easily trypsin‐digested cells compared to the undigestible ones. Furthermore, CD39 was determined to enhance cell invasion and suppress cell apoptosis but not affect cell proliferation. Moreover, CD39 could activate the TGF‐β/SMAD3 signalling pathway, whereas the expression of CD39 was negatively regulated by SMAD3 via recruitment of SETDB1 and adding H3K9me3 to the CD39 promoter in HCT116 cells. Overall, our study uncovered distinct gene signatures amongst different heterogeneities of colorectal cells and revealed the effect of CD39 on cell invasion and apoptosis, as well as determined the epigenetic role in regulating CD39 transcription.

## 1 Introduction

Tumour heterogeneity is one of the characteristics of malignant tumours. It means that during the growth process of tumours, after multiple divisions and proliferations, the daughter cells show changes in molecular biology or genes, resulting in differences in tumour growth rate, invasion ability, as well as sensitivity to drugs, prognosis and other aspects. Tumour heterogeneity comes from the heterogeneity of the distribution and effects of environmental factors and the randomness of gene mutations. In recent years, the tumour microenvironment has formed a complex regulatory relationship with targeted immunity, metabolic energy, drug resistance, and even microbial populations, providing a new perspective and strategy for human tumour treatment.

Cell culture can lead to a number of potential effects and changes, including that (1) Clonal drift: Prolonged cell culture can lead to clonal drift, that is, genetic variation in cell populations. This is due to mutations or epigenetic changes that may accumulate during cell division and passage; (2) Loss of differentiation ability: Some cell types have limited differentiation ability and may gradually lose their original differentiation potential in the process of continuous culture. A cell's ability to differentiate is affected by a variety of factors, such as accumulated genetic variation and cellular aging; (3) Accumulation of genetic mutations: Continuous culture may lead to the accumulation of genetic mutations. The genomic stability of cells can be affected, leading to the accumulation of genetic mutations that affect the cell's growth, function, and response; (4) Cell aging: Cells may experience aging in the process of long‐term culture. Cell aging occurs due to the accumulation of damage inside the cell and the limitation of the number of divisions, resulting in a decline in cell function and proliferation; (5) Loss of specific functions: Some cell types have specific functional or phenotypic characteristics, but these functions or characteristics may be gradually lost or changed in continuous culture.

In the process of cell culture in vitro, we usually find that the cells show signs of aging, which is that the passed cells are unable to stick to the wall. Experience tells us that the time it takes for trypsin to digest the adherent cells is important. If the digestion time is too short, enough cells cannot be obtained for passage, while if the digestion time is too long, a large number of senescent cells will also be digested. The substances released by the death and decomposition of these senescent cells will affect the function of those active cells, resulting in the failure of the recovery of the whole daughter cell line.

This project starts from the question of the global difference amongst cells undergoing multiple rounds of mitosis in one culture dish. RNA sequencing was conducted to investigate the transcriptome signatures of cells with different sensitivity responses to trypsin, and CD39 was picked up due to the significant down‐regulation in those senescent cells compared to those active ones. The function and regulatory mechanism of CD39 were further studied by molecular and cellular experiments. Our study reveals the expression changes and biological significance of CD39 in newborn and senescent cells. In addition to helping people make better use of tumour heterogeneity for cancer treatment, stable and excellent cell lines can also be obtained better in the passage of cell culture.

## 2 Materials and Methods

### 2.1 Cell Culture

Colorectal cancer HCT116 and HT29 cell lines obtained from the National Collection of Authenticated Cell Cultures (China) were cultured in Dulbecco's Modified Eagle Medium (DMEM) (Thermo Fisher Scientific, USA) and 20% foetal bovine serum (FBS) (Thermo Fisher Scientific).

For cell digestion, medium was removed, and cells with 80% density were simply washed by PBS twice. 1 mL 0.25% trypsin–EDTA (Thermo Fisher Scientific) was added into a 10 cm dish and uniformly dispersed for 0.5 min at 37°C, then quenched immediately by 4 mL regular medium. The suspended cells were harvested after centrifugation of 5 mL supernatant. 1 mL trypsin was added again to digest the rest of the cells for 2 min at 37°C, then the suspended cells were harvested as in the same procedure as above. The third round of 1 mL trypsin was added to digest the rest of the cells for 4 min at 37°C. These three batches of cells were used for the consequent experiments.

For cell count analysis, after subculture of 1, 2, 4, 8 and 12 h, attached cells were calculated by Vi‐CELL BLU (Beckman coulter, USA) following the removal of suspended cells.

CD39 siRNA (sense, 5′‐GGUUGUGAAUGUAAGCGAA‐3′, and antisense, 5′‐UUCGCUUACAUUCACAACC‐3′) was synthesised from Tsingke (China). The CD39 over‐expression plasmid was prepared by Sangon (China). Nucleotides were transfected into 1 × 10^6^ colorectal cells using the riboFECT Transfection Kit (Ribobio, China). TGFβ/Smad agonist SRI‐011381 (MedChemExpress, China) (10 μM 1 h) or TGFβ/Smad antagonist SIS3 (MedChemExpress) (5 μM 1 h) were treated with cells.

### 2.2 RNA Sequencing (RNA‐Seq)

Polysome‐associated RNA of at least 5 × 10^6^ cells was extracted with the FastPure Cell/Tissue Total RNA Isolation Kit V2 (Vazyme, China) according to the manufacturer's protocol. The extracted RNA was treated with DNase to remove genomic DNA contamination. Isolation of mRNA was based on the NEBNext PolyA mRNA Magnetic Isolation Module (New England Biolabs, USA) and the mRNA was further used with the NEB Next Ultra Directional RNA Library Prep Kit for Illumina (New England Biolabs) for sequencing library preparation. The libraries were quantified with Qubit 4.0 (Thermo Fisher Scientific) and the distribution of fragment sizes was determined with the Agilent Bioanalyser 2100 (Agilent, USA). Then, the libraries underwent Illumina sequencing with paired‐end 2 × 150 as the sequencing mode.

Raw reads were processed to obtain high‐quality clean reads by removing sequencing adapters and low‐quality reads using Cutadapt v1.18 (using default parameters except ‐‐max‐n 0) and Trimmomatic v0.35 (using default parameters except SLIDINGWINDOW:4:15 LEADING:10 TRAILING:10). Then, FastQC (using default parameters) was used to ensure high quality of the clean reads (https://www.bioinformatics.babraham.ac.uk/projects/fastqc/). After that, the clean reads were mapped to the mouse genome (assembly GRCm38) with HISAT2 v2.1.0 (using default parameters except ‐‐rna‐strandness RF ‐‐dta). Gene expression levels were measured with FPKM (fragments per kilobase of exon per million fragments mapped) by StringTie v1.3.4 (using default parameters except ‐e ‐‐rf). The R package, edgeR, was employed to identify differentially expressed genes (DEGs). Those genes with *p*‐values < 0.05 and |log_2_(Fold Change)| > 1 were considered differentially expressed and used for downstream analysis. Human gene annotation files (GRCh38) were retrieved from the Ensembl genome browser 96 database (http://www.ensembl.org/index.html).

### 2.3 Immunofluorescence (IF)

Cells were fixed within 4% solution of paraformaldehyde and washed by PBS, then permeabilised with 0.1% Triton‐X‐100 and blocked with 0.5% horse serum in PBS. Immunostainings of samples were performed using antibodies of CD39 (1:250, Cat. No. 19229‐1‐AP, Proteintech, China), SMAD3 (1:500, Cat. No. 66516‐1‐Ig, Proteintech), p‐SMAD3 (1:300, Cat. No. ab52903, Abcam, USA) overnight at 4°C. After washing with PBS for four times (5 min per time), the donkey anti‐rabbit secondary antibody (1:20,000; Proteintech) and goat anti‐mouse secondary antibody (1:20,000; Proteintech) were used to incubate for 30 min at room temperature, following further incubation with DAPI for 15 min, and washed by PBS for four times. After drying in the air, slices were dropped with coated with mounting medium and coated with cover glasses. The positive staining was captured by LSM800 Confocal Laser Scanning Microscopy (Zeiss, Germany).

### 2.4 Western Blot (WB)

5 × 10^6^ cells with RIPA lys3is buffer according to the manufacturer's protocol. The protein concentrations were quantified with Bradford protein assay reagent (Bio‐Rad, USA). The samples were resolved by 8%–12% of SDS‐PAGE and transferred to nitrocellulose membranes (Millipore, USA). To show the level changes of protein CD39, Ki67, MCM, AFP, CEA, BCL‐2, Caspase3, CD73, Collagen I, Collagen III, and β‐Actin, aliquots of proteins were immunoblotted with primary antibodies (Ki67: 1:2000, Cat. No. 27309‐1‐AP, Proteintech; MCM: 1:2000, Cat. No. 67071‐1‐Ig, Proteintech; AFP: 1:2000, Cat. No. 14550‐1‐AP, Proteintech; CEA: 1:2000, Cat. No. 10421‐1‐AP, Proteintech; BCL‐2: 1:2000, Cat. No. 12789‐1‐AP, Proteintech; Caspase3: 1:2000, Cat. No. 66470‐2‐Ig, Proteintech; CD73: 1:2000, Cat. No. 12231‐1‐AP, Proteintech; Collagen I: 1:2000, Cat. No. 14695‐1‐AP, Proteintech; Collagen III: Cat. No. 22734‐1‐AP, Proteintech; β‐Actin: 1:5000, Cat. No. 81115‐1‐RR, Proteintech; SMAD3: 1:2000, Proteintech; SETDB1: 1:2000, Proteintech; SMAD4: 1:2000, Cat. No. 10231‐1‐AP, Proteintech; TRIM28: 1:2000, Cat. No. 15202‐1‐AP, Proteintech; PAF1: 1:2000, Cat. No. 15441‐1‐AP, Proteintech; HRP‐conjugated streptavidin: 1:5000. Cat. No. SA00001‐0, Proteintech) at 4°C overnight and then incubated with secondary antibodies at room temperature for 1 h. The membranes were developed by Hypersensitive ECL Chemiluminescence Kit (Proteintech). The protein expression levels were normalised with respect to β‐actin. Western blot images were scanned by ChemiScope 6000EXP System (Clinx Science Instruments Co. Ltd., China), then analysed using the Image J software (Bio‐Rad).

### 2.5 Flow Cytometry (FC)

Digested HCT116 and HT29 cells with different conditions of trypsin were washed with PBS twice, and mixed with 80% ethanol at −20°C overnight, then washed with PBS twice again, resuspended in 500 μL PBS, and mixed with 5 μL CD39 antibody (Cat. No. 561444, BD Biosciences, USA) combined with 5 μL Ki67 (Cat. No. 570922, BD Biosciences), AFP (Cat. No. 563002, BD Biosciences) or PI (MedChemExpress, China) gently, then kept in the dark for 15 min. After that, cells were centrifuged at 200 × *g* for 5 min, the supernatant was removed, and cells were resuspended in 1 mL PBS. FITC (CD39), PE (Ki67, AFP) and FL2 (PI) channels were selected for cell apoptosis assay using Attune NxT Flow Cytometer (Thermo Fisher Scientific).

### 2.6 Chromatin Immunoprecipitation (ChIP)

The main experimental process of the ChIP assay was referred to in our previous studies [1, 2, 3]. 1 μg ChIP grade antibodies against SMAD3 (Cat. No. NBP2‐20411, Novus Biologicals, USA), SETDB1 (Cat. No. 11231‐1‐AP, Proteintech), H3K9me3 (Cat. No. MABI0319, Proteintech), H3K9ac (Cat. No. 9649, CST), p300 (Cat. No. 54062, CST), RNA pol II (Cat. No. NB200‐598, Novus Biologicals) and IgG (Cat. No. 30000‐0‐AP, Proteintech) were used in this study.

### 2.7 Quantitative Polymerase Chain Reaction (qPCR)

Template cDNA was used to perform PCR within a 20 μL reaction mixture containing specific primers (Tsingke) using BeyoFast SYBR Green qPCR Mix (2×) (Cat. No. D7260, Beyotime). The primer sequence of CD39 was F: 5′‐GTTCATACTTGGTTGAACCTAA‐3′; R: 5′‐AGGAGACTGCAGAATGAAGACCT‐3′. The annealing temperature was 58°C.

### 2.8 Pull Down Assay

Biotinylated DNA probe against the promoter of CD39 was synthesised by Tsingke. 5 × 10^6^ HCT116 cells were lysated using 1 mL ice‐cold RIPA buffer (0.5% NP40 as the detergent) for 30 min with occasional vortexing. After 12,000 × *g* centrifugation for 30 min, the protein supernatant was transferred into a new Eppendorf tube. 200 μmol probes were pre‐incubated with 20 μL Streptavidin Magbeads (YEASEN, China) at 4°C for 30 min (the amount for one experiment). Then cell lysate and probe/beads were mixed at 4°C for 2 h with slow rotation. After washing accordingly, 100 μL Elution buffer was added to the harvested protein in a boiled water bath for 10 min. Supernatant was transferred into another new Eppendorf tube for mass spectrometry after 12,000 × *g* centrifugation. HPLC‐MS/MS method provided by OE Biotech (China) was used to identify the binding proteins on SMAD3 and semi‐quantify the difference amongst different groups.

### 2.9 Statistical Analysis

All experimental data were processed and analysed using SPSS 22.0 statistical software (IBM Corp., USA). The measurement data were expressed as mean ± standard deviation. One‐way ANOVA was used for comparison between multiple groups. The *p*‐value less than 0.05 was considered statistical significance.

## 3 Results

### 3.1 Loss of CD39 Expression in Colorectal Cancer Cells by Passaging In Vitro

**FIGURE 1:**
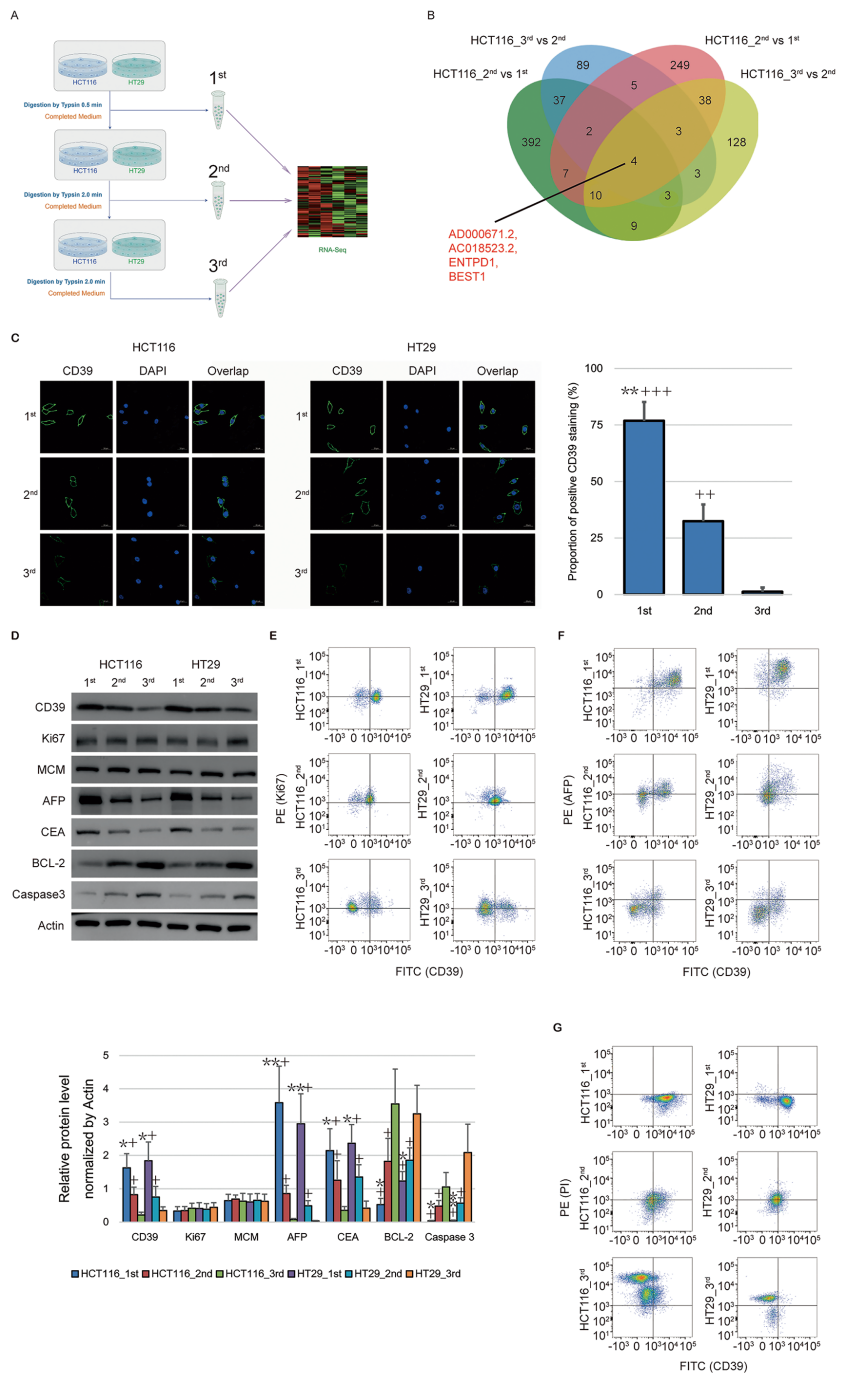
CD39 is a marker of easily digestible colorectal cancer cells. (A) Graphical representation shows the colorectal cancer HCT116 and HT29 cells digested by trypsin with different timing conditions were processed for RNA sequencing. (B) Venn diagram shows the intersection of DEGs amongst these different groups of cell. ENTPD1 (CD39) is one of the intersection of highly expressed genes in the 1st vs. 2nd round, as well as 2nd vs. 3rd round digested HCT116 and HT29 cells. (C) IF assay and statistical analysis show the significantly differential expression of CD39 in different rounds of HCT116 and HT29 cells. (D) WB assay show and statistical analysis show the significantly differential expression of CD39 in different rounds of HCT116 and HT29 cells. Moreover, the differential expression of AFP/CEA and BCL‐2/Caspase 3 indicate the distinct cell invasion ability as well as apoptosis. (E–G) Flow cytometric assay show the co‐expression of CD39 and Ki67 (E), AFP (F) or PI (G). “*”, “**”: Statistical significance compared to 2nd round cells with *p* value less than 0.05 and 0.01; “+”, “++”, “+++”: Statistical significance compared to 3rd round cells with *p* value less than 0.05, 0.01 and 0.001. AFP, alpha foetal protein; BCL‐2, BCL2 apoptosis regulator; CEA, carcinoembryonic antigen; ENTPD1/CD39, ectonucleoside triphosphate diphosphohydrolase 1; FITC, fluorescein 5‐isothiocyanate; Ki67, marker of proliferation Ki‐67; MCM, minichromosome maintenance complex component 3; PE, phycoerythrin; PI, propidium iodide.

We collected three rounds of colorectal cancer HCT116 and HT29 cells according to distinct conditions of trypsin digestion (0.5, 2, 2 min) in order. These cells, indicating distinct adhesion capacity, were further processed by RNA‐seq (Figure 1A). We noticed that Ectonucleoside triphosphate diphosphohydrolase 1 (Entpd1) (encoding protein is CD39) was highly expressed (log_2_ Fold change > 1, *p* < 0.05) in the first cells compared to the later ones to be digested of HCT116 and HT29 cells (Figure 1B, Table S1). IF (Figure 1C) and WB (Figure 1D) assays both verified the expression signature of CD39 in different batches of colorectal cancer cells of the current system.

Then, we investigated the cell proliferation, invasion, and apoptosis of these different batches of colorectal cancer cells. Herein, we directly studied the freshly digested cells using WB and flow cytometric assays rather than the regular experiments including CCK‐8 and transwell assays. We observed that the no significant difference in Ki‐67 and MCM expression indicated no change in the ability of cell growth amongst these three batches of cells (Figure 1D), whereas the elevated expression of AFP and ECA suggested the robust invasion ability of the first cells compared to the later ones to be digested (Figure 1D). Additionally, the cells digested later also showed more potential apoptosis compared to the cells digested earlier by the expression of BCL‐2 and Caspase 3 (Figure 1D). Consistently, the flow cytometric assay confirmed that different subpopulations of HCT116 and HT29 cells isolated by the expression of CD39 could also distinguish between the phenotypes of cell invasion and apoptosis (Figure 1E–H).

Now we determined that tumour heterogeneity by the sensitivity to trypsin showed significantly differential phenotypes of cell invasion and apoptosis, and CD39 was one of the important biomarkers for the identification of this tumour heterogeneity of colorectal cancer cells in vitro in our research system.

### 3.2 Cell Invasion and Apoptosis of Colorectal Cancer Cells Affected by CD39

**FIGURE 2:**
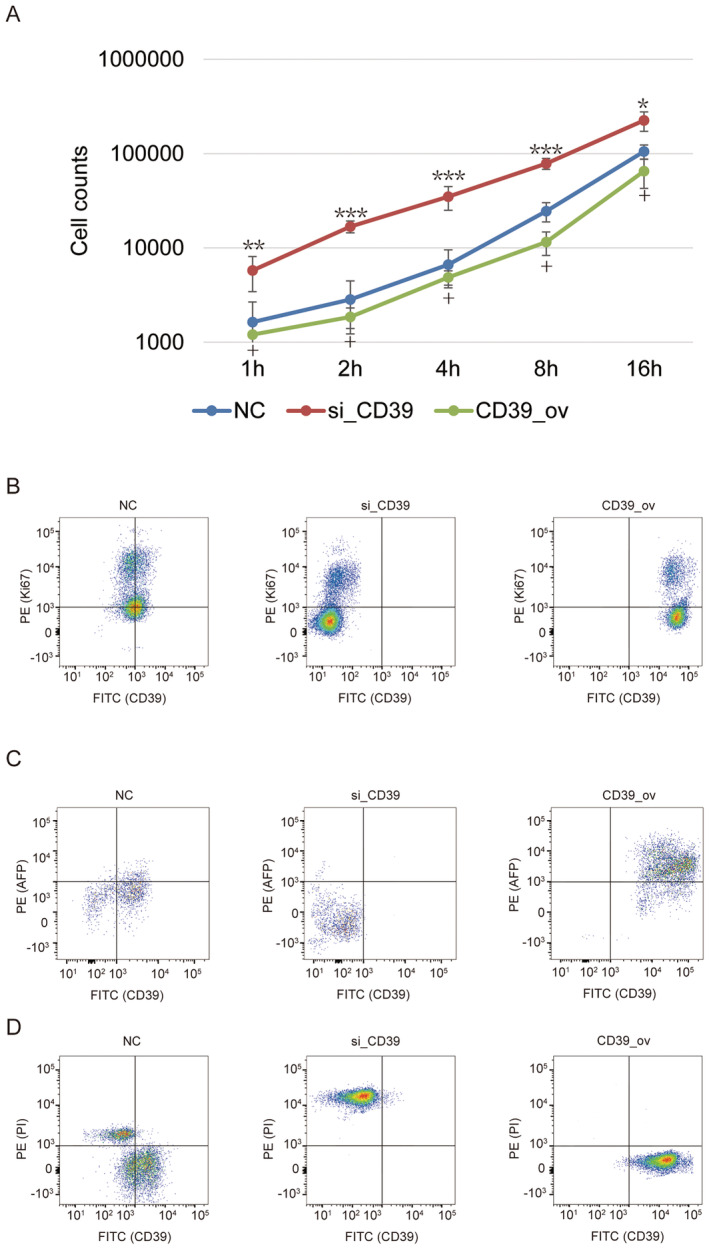
CD39 modulates cell invasion and apoptosis in colorectal cancer cells. (A) Cell adhesion assay show the ability of cell adhesion by CD39 knockdown or over‐expression. (B–D) Flow cytometric assay show the expression of Ki67 (B), AFP (C) or PI (D) by CD39 knockdown or over‐expression. “*”, “**”, “***”: Statistical significance compared to 2nd round cells with *p* value less than 0.05, 0.01 and 0.001; “+”: Statistical significance compared to 3rd round cells with *p* value less than 0.05.

**FIGURE 3:**
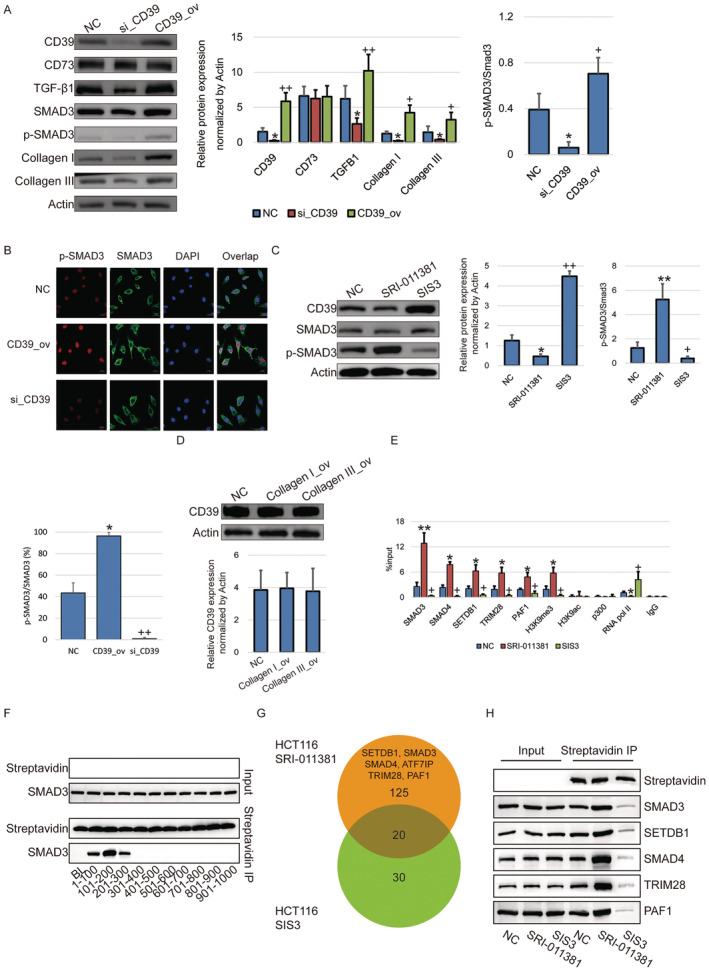
The negative feedback of CD39 transcription regulated by SMAD3. (A) WB assay and statistical analysis show the activity of TGF‐β1/SMAD3 signalling pathway and the expression of Collagen I/III affected in HCT116 cells by CD39 knockdown or over‐expression. (B) IF assay show the subcellular localisation of phosphorylated SMAD3 in HCT116 cells. Proportion (P) and brightness (B) of cells with positive p‐SMAD3 and SMAD3 staining were compiled. Brightness value was considered as 0 to 5. $\text{Ratio}=\left(\sum_{1}^{10}\frac{P\left(p-\text{SMAD}3\right)\times B\left(p-\text{SMAD}3\right)}{P\left(\text{SMAD}3\right)\times B\left(\text{SMAD}3\right)}\right)÷10\times 100\%$. (C) WB assay and statistical analysis verify the phosphorylation of SMAD3 in HCT116 cells treated with SMAD3 agonist (SRI‐001381) and antagonist (SIS3). (D) WB assay and statistical analysis show the expression of CD39 in HCT116 cells by Collagen I or III over‐expression. (E) ChIP‐qPCR assay show the occupancies of SMAD3, SMAD4, TIM28, PAF1, RNA pol II, SETDB1, H3K9me3 and H3K9ac and p300 on promoter of CD39 in HCT116 cells treated with SMAD3 agonist (SRI‐001381) and antagonist (SIS3). (F) CD39 promoter truncation experiments show the binding affinity of SMAD3 on different truncated promoter regions pulled down by streptavidin. (G) Venn diagram show the binding proteins on CD39 promoter by mass spectrum. SMAD3 agonist can efficiently recruit SETDB1, SMAD3, SMAD4, ATF7IP, TRIM28 and PAF1 to form silencing complex compared with SMAD3 antagonist. (H) WB assay show the SMAD3, SMAD4, SETDB1, TRIM28 and PAF1 as the components in the co‐repressor complex at CD39 promoter in streptavidin pull down product of HCT116 cells with SMAD3 agonist (SRI‐001381) treatment compare to SMAD3 antagonist (SIS3).

Since the certain correlation between the expression of CD39 and the phenotypes of cell invasion and apoptosis of HCT116 and HT29 cells was found, artificially intervening in CD39 expression in colorectal cells was performed. The negative regulation of the expression of collagen I and III (Figure 3A) as well as cell adhesion ability (Figure 2A) both determined that CD39 was not conducive to cell adhesion. Moreover, no significant change in Ki67 expression was observed by CD39 knockdown or over‐expression (Figure 2B). The expression of AFP was significantly up‐regulated by CD39 over‐expression, whereas down‐regulated by CD39 knockdown (Figure 2C), indicating that CD39 could enhance the cell invasion of colorectal cancer cells. Likewise, the cell apoptosis assay also determined that elevated CD39 could avoid cells from apoptosis (Figure 2D). These phenotypes of cell proliferation, invasion and apoptosis strengthened the causal connection between CD39 and cell viability.

### 3.3 Negative Feedback Between CD39 and SMAD3 via Epigenetic Manner

CD39/CD73 was reported to contribute to extracellular ATP hydrolysis in multiple physiological and pathological processes in various diseases [4]. Previous studies indicated that blockade or silencing of CD39 could suppress the activation of the adenosine A2A and adenosine A2B receptors and the TGF‐β/Smad3 pathway [5]. Our observations suggested that CD39 knockdown indeed resulted in the failure of entry into the nucleus of the dephosphorylated SMAD3 (Figure 3A,B). SMAD could be associated with a variety of transcription factors, so each step of the SMAD regulatory process could interact with proteins in other pathways to form signal crosstalk, thereby achieving transcriptional activation or suppression of effecting genes. This also led to extensive even opposite regulatory effects of SMAD in different cell physiological states [6, 7, 8].

We questioned the phenotype of the spontaneously weakened CD39 expression along with cell passaging. To this end, the activity of the TGF‐β/Smad3 pathway in HCT116 cells was intervened (Figure 3C), and unexpectedly, the expression of CD39 was remarkably compromised by the TGF‐β/Smad agonist (SRI‐011381) while boosted by the TGF‐β/Smad inhibitor (SIS3) (Figure 3C). On the other hand, Collagen I or III failed to affect the expression of CD39 (Figure 3D).

Furthermore, the epigenetic regulation by SMAD3 on the promoter of CD39 was investigated by ChIP‐qPCR. It was notable that the occupancy of RNA pol II was compromised along with SMAD3 gathering on the CD39 promoter. Moreover, the enrichment of H3K9me3, the transcriptional repression hallmark, and the occupancy of histone methyltransferase SETDB1 appeared to be robust after SMAD3 was recruited on the CD39 promoter (Figure 3E). On the contrary, H3K9ac, as a sign of transcriptional activation, and p300 showed low abundance on the CD39 promoter compared to IgG, and no significant change by SMAD3 binding (Figure 3E). Moreover, we separately split the 1000 bp promoter sequence upstream of CD39 into 20 segments, then generated each 100 bp segment labelled with biotin. After incubation with the nuclear lysate, the synthesised sequences were captured by streptavidin, and the SMAD3 protein was detected. We observed that SMAD3 mainly bound with the promoter region at 300 bp upstream of ATG (Figure 3F).

Finally, binding proteins on the CD39 promoter pulled down by biotin‐labelled DNA probes were analysed by mass spectrometry. SETDB1, SMAD3, SMAD4, TRIM28, and PAF1 were found in HCT116 cells treated with agonist compared to antagonist (Figure 3G, Table S2). ChIP‐qPCR (Figure 3E) and WB (Figure 3H) further validated these co‐repressors as the crucial components within this complex by streptavidin pull‐down products, implying that the repressive complex was recruited and formed at the promoter of CD39 after the TGF‐β/Smad3 pathway was activated.

The data above suggested that CD39 could activate the TGF‐β/SMAD3 signalling pathway, while SMAD3 in turn functioned in gene silencing via recruitment of SETDB1 for H3K9me3 on the CD39 promoter.

## 4 Discussion

Trypsin, as an ideal enzyme for digesting extensive and complex proteomes, is utilised in mass spectrometry, tissue dissociation and cell culture [9]. Trypsin acts on peptide bonds linked to lysine or arginine, removes intercellular mucins and glycoproteins, affects adhesion between cells and substrates, and thus causes cell separation. The commonly used trypsin solution concentration is 0.25%; the higher the trypsin solution concentration, the stronger the effect, but beyond a certain limit it will damage cells. Meanwhile, the loss of cell surface proteins due to excessive enzymatic degradation may influence the analysis of surface markers and reduce cell viability, especially that of stem cells [10]. In practical application, the commercial trypsin–EDTA (0.25%) is usually further diluted 10 or 20 times before being used. For those cells, such as keratinocytes, fibroblasts, neurons and glial cells, showing robust cell adhesion, it is necessary to increase the timing and concentration of trypsin digestion against the resistance to trypsin cleavage by the abundant fibronectin and glycoproteins on the surface membrane [11]. Incubation time actually has a greater effect on cell damage than trypsin concentration [12]. Therefore, in the process of in vitro cell experiment, the distinct treatment conditions of trypsin digestion and passage in different study groups will have a profound impact on cell function, which should be standardised by corresponding literatures.

Our data has shown that distinct trypsin‐digested conditions may determine and distinguish the different statuses of cells due to the global transcriptome (Figure 1B). We believe that cell fate screened by trypsin is supposed to be conserved and applicable to other types of cells beyond HCT116 and HT29 cells, but a part of signalling pathway activity and gene expression has appeared to be different depending on the cell lineages and types. Our RNA sequence has indicated CD39 as a candidate gene under the relatively strict conditions. The main highlight of this study is to find CD39 and demonstrate its close relationship with the cell invasion ability of colorectal cancer cells. CD39, in conjunction with CD73, mainly functions on creating an immunosuppressive microenvironment for cancer via extracellular adenosine triphosphate (ATP) to ADP, AMP and adenosine conversion [4]. Nevertheless, the so‐called immunosuppressive microenvironment is out of the discussion scope in the culture system of colorectal cancer cells in vitro. Previous studies have suggested that CD39 is positively correlated with cell invasion and metastasis [13, 14, 15] via the adenosine/A2B receptor signalling pathway in the community of cancer cells [16]. Therefore, although the biological mechanism of CD39 inside the colorectal cancer cells remains to be further clarified, we only construct the connection between CD39 and cell invasion (Figures 1D–G and 2), but avoid the duplication of similar efforts in current research.

In turn, as the second highlight of this study, we have addressed a novel epigenetic regulation of CD39 expression, and we have uncovered that SMAD3 plays a suppressive role in CD39 transcription through recruiting and forming a silencing complex (Figure 3). Whether SMAD activates or inhibits transcription depends on a number of factors, including other relevant transcription factors/coregulators, target genes, the physiological state of the cell, and the composition of the SMAD tripolymer. The variety of transcription factors/coregulators associated with SMAD also leads to a wide range of regulatory effects of SMAD, which may even have opposite effects in different cell physiological states. For example, the activated Receptor regulated‐SMAD complex recruits histone modifying enzymes, resulting in chromatin remodelling. Recruitment of acetyltransferase p300 typically acts as a transcriptional coactivator of the SMAD complex by elevation of genomic H3K9ac, whereas the recruitment of methyltransferase SETDB1 can induce H3K9me3, thereby inhibiting transcription, and SMAD‐mediated recruitment of histone deacetylases also causes histone deacetylation [6]. This dual effect shows that the TGF‐β pathway has two opposite effects. In normal cells and in the early stages of cancer development, it acts as a tumour suppressor, inhibiting cell proliferation, promoting the maintenance of genomic stability, and stimulating abnormal cell apoptosis [17]. However, in the later stage of tumour development, TGF‐β tumour inhibition is destroyed and instead becomes a tumour‐ promoting factor, which can induce epithelial‐mesenchymal transformation (EMT), promote tumour cell invasion and metastasis, and has an immunosuppressive effect in the tumour microenvironment, and promotes angiogenesis [8]. This transformation can occur because during the development of tumours, the cell genome continuously changes at the DNA and epigenetic modification levels, resulting in changes in the regulatory network in the cell.

In summary, we propose some suggestions for trypsin digestion in cell culture and passage for peer reference and briefly reveal a novel epigenetic regulation of an important marker CD39 in colorectal cancer cells.

## Author Contributions

**Xiaosong Li:** investigation (equal), methodology (equal). **Yifen Shen:** investigation (equal), methodology (equal). **Jianhua Lang:** resources (equal), software (equal). **Jianzhong Wu:** resources (equal), software (equal). **Zhenhai Qian:** resources (equal), software (equal). **Genhai Shen:** resources (equal), software (equal). **Yihang Shen:** conceptualization (lead), data curation (lead), formal analysis (lead), funding acquisition (lead), investigation (lead), project administration (lead), resources (equal), software (equal), supervision (lead), validation (lead), visualization (lead), writing – original draft (lead), writing – review and editing (lead).

## Conflicts of Interest

The authors declare no conflicts of interest.

## Supporting information


**Table S1.** Differentially expressed genes (DEGs) compared amongst colorectal cancer cell lines by the trypsin digestion with different conditions. List A to C: DEGs of HCT116 compared between 2nd digestion to 1st digestion. List D to F: DEGs of HCT116 compared between 3rd digestion to 2nd digestion. List G to I: DEGs of HCT29 compared between 2nd digestion to 1st digestion. List J to L: DEGs of HCT29 compared between 3rd digestion to 2nd digestion. Gene with fold change more than 2 or less than 0.5 and *p* value less than 0.05 is considered as a DEG.

**Table S2.** Binding proteomics of CD39 promoter pulled down by biotin‐labelled DNA probes characterised by mass spectrometry. Line 1 to 148 are the candidate proteins of two replications of HCT116 cells treated with TGFβ/Smad agonist SRI‐011381. Line 149 to 199 are the candidate proteins of two replications of HCT116 cells treated with TGFβ/Smad antagonist SIS3.

## Data Availability

Data will be made available on request.
