# Supplementary material for: CD39 Contributes to the Ability of Cell Invasion in Heterogeneity of Colorectal Cancer
Source: J Cell Mol Med. 2025 Mar 7;29(5):e70486. doi: 10.1111/jcmm.70486 (PMC11886887; doi:10.1111/jcmm.70486)
Supplement: Supplementary file 1 — Table S1. Differentially expressed genes (DEGs) compared amongst colorectal cancer cell lines by the trypsin digestion with different conditions. List A to C: DEGs of HCT116 compared between 2nd digestion to 1st digestion. List D to F: DEGs of HCT116 compared between 3rd digestion to 2nd digestion. List G to I: DEGs of HCT29 compared between 2nd digestion to 1st digestion. List J to L: DEGs of HCT29 compared between 3rd digestion to 2nd digestion. Gene with fold change more than 2 or less than 0.5 and p value less than 0.05 is considered as a DEG. [file JCMM-29-e70486-s001.zip › jcmm70486-sup-0001-TableS1.docx]

| **GeneName** | **116_2ndvs1st_FC(1.5/0.5)** | **pvalue** | **GeneName** | **116_3rdvs2nd_FC(1.5/0.5)** | **pvalue** | **GeneName** | **29_2ndvs1st_FC(1.5/0.5)** | **pvalue** | **GeneName** | **29_3rdvs2nd_FC(1.5/0.5)** | **pvalue** |
| --- | --- | --- | --- | --- | --- | --- | --- | --- | --- | --- | --- |
| WFDC10B | 16.20025 | 0.000289319 | AC092143.1 | 97.64915 | 1.45986E-27 | AP003419.1 | 68.15095 | 2.52529E-14 | TRIM39-RPP21 | 67.65595 | 4.03525E-19 |
| SMCP | 13.7097 | 0.000174428 | SPEGNB | 23.9159 | 0.000232597 | BEST1 | 25.53395959 | 0 | H3C11 | 24.0992 | 6.45196E-05 |
| PGLYRP3 | 12.5073 | 2.23051E-05 | SYNGR4 | 20.2852 | 3.44466E-06 | GNG3 | 22.16525 | 7.49444E-07 | NPPA | 21.9401 | 9.15088E-07 |
| SMIM36 | 9.050677176 | 0.00037842 | PAGE2B | 18.87895 | 0.000223268 | NPHP3-ACAD11 | 10.80298821 | 0.000154046 | TMCO2 | 20.42145 | 2.15007E-05 |
| AC012488.2 | 9.000094091 | 0.001153728 | PPY | 14.4353 | 0.00046933 | ITIH4 | 9.701429328 | 0.003951912 | LRRC70 | 19.2657 | 3.02323E-11 |
| PINX1 | 6.696476713 | 0.000408067 | C1QTNF3-AMACR | 13.59151971 | 5.42197E-10 | RHOXF1 | 8.213793877 | 0.000375206 | AL133500.1 | 16.70788166 | 1.67988E-10 |
| UTP14C | 5.369590436 | 0.000214768 | TMPRSS11E | 12.54505 | 8.74463E-08 | C2 | 6.59118392 | 5.51686E-05 | BORCS7-ASMT | 13.919 | 2.28867E-10 |
| SPEM2 | 5.049893999 | 0.000594582 | AL445685.3 | 9.61530301 | 4.7193E-14 | RSPH14 | 6.022631498 | 0.000375713 | FAM220A | 8.137636045 | 2.61956E-07 |
| AC005833.1 | 4.951389265 | 0.004074458 | MZB1 | 6.53696708 | 0.026273745 | AL928654.3 | 5.758658583 | 6.8875E-05 | TBC1D3C | 7.957660908 | 5.06694E-06 |
| C15orf48 | 4.885100868 | 0.000496231 | AC009070.1 | 6.302623774 | 0.000106637 | AP001273.2 | 5.228800965 | 0.000305 | CORT | 7.710665605 | 0.000107993 |
| SMIM6 | 4.796959914 | 0.001377245 | ASDURF | 6.032872505 | 0.001255854 | AC023055.1 | 5.21130371 | 0.007151577 | H2AC12 | 7.184155299 | 0.004468064 |
| URGCP-MRPS24 | 4.764870888 | 0.030946689 | AL160269.1 | 5.967804105 | 8.48154E-11 | CORO7-PAM16 | 5.145374841 | 0.000974385 | TGFBR3L | 7.150264448 | 0.000105414 |
| APOC1 | 4.590763866 | 0.008712645 | AC011479.1 | 5.962888865 | 0.000158845 | AC138894.1 | 5.12665505 | 0.022313941 | UGT1A5 | 6.595902445 | 9.78778E-08 |
| CYP2C9 | 4.484789807 | 0.002720262 | AC003002.2 | 5.920599974 | 0.010758723 | KCNAB3 | 4.955032441 | 0.001048616 | RTBDN | 6.179526997 | 0.001332848 |
| SEC16B | 4.483423392 | 0.001233483 | TMEM89 | 5.035808685 | 0.01498548 | CENPS-CORT | 4.683572748 | 0.000416363 | TH | 6.011489791 | 0.002557813 |
| AL358113.1 | 3.877556841 | 1.12855E-05 | AC091057.6 | 4.878035215 | 1.84677E-06 | FAM209A | 3.94181648 | 0.038402299 | AGXT2 | 5.916758513 | 0.000187417 |
| CMTM2 | 3.724130026 | 0.006885343 | PLAAT2 | 4.806460913 | 0.019728909 | AP002884.2 | 3.91240113 | 0.047549741 | LY6G6C | 5.556920807 | 8.39797E-05 |
| AP000311.1 | 3.69944682 | 0.007713388 | STPG3 | 4.382053362 | 0.010260727 | TSACC | 3.794499533 | 0.028396312 | GZMB | 5.476597011 | 0.002984689 |
| RPL36A-HNRNPH2 | 3.588987809 | 0.001632122 | BHLHB9 | 4.282896648 | 0.007287721 | AC036214.3 | 3.742831783 | 0.021289053 | TREX1 | 5.257036062 | 4.2245E-07 |
| CLCNKA | 3.494258563 | 0.046043914 | SSX1 | 4.24827249 | 0.002368072 | ATP1A4 | 3.727091755 | 0.006157271 | RPA4 | 5.24474893 | 0.000182357 |
| AC013477.2 | 3.493921545 | 0.000316832 | ROPN1L | 4.192304257 | 0.001552104 | TMIE | 3.439030225 | 0.000625717 | ABHD14A-ACY1 | 4.772735754 | 1.31825E-05 |
| TEN1 | 3.454785794 | 0.000318526 | AL603832.3 | 4.132882677 | 0.031883361 | AL022238.3 | 3.280737242 | 3.66812E-08 | TTYH1 | 4.718190881 | 0.000633298 |
| HSD17B3 | 3.354108522 | 0.01944705 | ATP6V1G2-DDX39B | 4.070803085 | 2.13178E-05 | TNFRSF19 | 3.193451897 | 1.56137E-05 | AC091167.2 | 4.515879131 | 0.016145132 |
| TAS2R19 | 3.336836061 | 0.014898189 | AMHR2 | 4.019994347 | 0.030696159 | AP002990.1 | 3.154352833 | 0.010863302 | C6orf15 | 4.412592902 | 0.001665425 |
| CA9 | 3.298855458 | 0.000428451 | AC000120.4 | 3.819943698 | 0.01437194 | MDH1B | 3.128935164 | 0.004553973 | KRTAP5-2 | 4.249316443 | 1.72882E-05 |
| SMCO2 | 3.259769669 | 0.006551197 | LCE1C | 3.781936146 | 0.040902676 | AD000671.2 | 3.086288594 | 6.21909E-05 | SCHIP1 | 4.139042849 | 0.000611851 |
| CPXM1 | 3.208332082 | 0.003285571 | DIO2 | 3.396990214 | 0.003840223 | FITM1 | 3.052487507 | 0.023491075 | ATOH7 | 3.997802543 | 0.002280305 |
| MROH7-TTC4 | 3.175527462 | 0.004066631 | LRRC32 | 3.318853405 | 0.035172464 | EVPLL | 2.835811309 | 0.011579851 | H1-4 | 3.97762091 | 7.59068E-07 |
| RPE65 | 3.123792416 | 0.004209647 | HRG | 3.3075741 | 0.007300662 | TVP23C-CDRT4 | 2.775087762 | 0.025418266 | HAO2 | 3.937070903 | 0.012840692 |
| ANKRD2 | 3.026345808 | 0.015549778 | H2BC13 | 3.274205295 | 0.041420318 | SLC51A | 2.771455511 | 0.009280153 | AL109811.3 | 3.915338237 | 0.00198936 |
| KLKB1 | 3.016367768 | 0.013290707 | TEX13B | 3.242829555 | 0.029292836 | AC087721.2 | 2.648759366 | 5.77413E-05 | TAS2R3 | 3.910126403 | 0.001269271 |
| AC018523.2 | 3.015648688 | 0.01412434 | DDX25 | 3.240216183 | 0.023761748 | NRN1 | 2.544752176 | 0.012986555 | LRRIQ4 | 3.889859902 | 0.021306445 |
| C9orf24 | 2.974141583 | 0.012384092 | AC025283.2 | 3.233711067 | 0.036510346 | FAM186B | 2.533876533 | 0.038263093 | POLN | 3.79097733 | 0.00101799 |
| CXCL17 | 2.972300202 | 0.036883139 | AC068775.1 | 3.195437888 | 2.14041E-06 | AC011473.4 | 2.454230985 | 0.01577746 | HBE1 | 3.770944721 | 0.01678921 |
| AL136295.4 | 2.917981089 | 0.000277166 | GP1BB | 3.083346723 | 0.006594934 | AL391650.1 | 2.394904412 | 0.03318559 | AC007731.4 | 3.683469279 | 0.042746649 |
| AC068631.2 | 2.906032597 | 0.006421359 | CAMK1G | 2.993843572 | 0.013874964 | PLA1A | 2.371329226 | 0.002434812 | ARMC12 | 3.592971809 | 0.000640885 |
| VSIG2 | 2.892532383 | 0.042445428 | BEST1 | 2.976392043 | 3.48039E-06 | AP001931.2 | 2.327580889 | 0.017777324 | AC009163.5 | 3.590854383 | 0.012191549 |
| ZFPM2 | 2.849341699 | 0.015936078 | ZNF454 | 2.972696553 | 0.005508117 | PTGES3L | 2.28444424 | 0.00651041 | EVL | 3.526973967 | 0.004562649 |
| KRT14 | 2.834266876 | 0.041715973 | SOX7 | 2.932398454 | 0.000555862 | MATR3 | 2.260174542 | 3.37965E-07 | NEXN | 3.487350584 | 0.018745 |
| CNTD1 | 2.833603637 | 0.01391855 | ACTA1 | 2.858196129 | 0.006925823 | DUS4L-BCAP29 | 2.259547025 | 6.36007E-11 | H4C2 | 3.461767558 | 0.026884067 |
| PDZD2 | 2.808703013 | 0.026857537 | GADD45G | 2.814470467 | 0.01376026 | NPIPB2 | 2.239283923 | 0.01997362 | AC009690.1 | 3.426937999 | 8.80332E-05 |
| VPREB3 | 2.807125161 | 0.025031024 | IL4I1 | 2.813220687 | 0.012308045 | APOBEC1 | 2.219592925 | 0.044010707 | TAS2R46 | 3.396372658 | 0.04628063 |
| KCNJ10 | 2.797889598 | 0.04261021 | LDLRAD4 | 2.782599147 | 0.037489619 | HAS2 | 2.217071669 | 0.005280212 | H1-5 | 3.384279242 | 0.004155011 |
| TMEM253 | 2.735997384 | 0.024072949 | MAP1LC3C | 2.775773493 | 0.018960916 | OR13A1 | 2.211675233 | 0.036130111 | FGFBP1 | 3.35212847 | 0.000605518 |
| ZMYND12 | 2.672947548 | 0.012727493 | AC007192.1 | 2.660190775 | 2.74038E-05 | GIMAP2 | 2.19065507 | 0.008134366 | AL031777.2 | 3.291652317 | 1.0459E-06 |
| GLIS1 | 2.594073452 | 0.004513257 | FOXE3 | 2.575221882 | 0.037610309 | PRRT1 | 2.11961446 | 0.027248157 | ANKRD37 | 3.277978792 | 5.46901E-07 |
| SPATA21 | 2.593112007 | 0.005152169 | AC005324.3 | 2.520694419 | 0.002701235 | SLC30A2 | 2.119313714 | 0.04418018 | DLEC1 | 3.188011005 | 0.00045972 |
| TVP23C-CDRT4 | 2.582566463 | 0.002358126 | AC025165.6 | 2.518608475 | 0.006025655 | MIA | 2.110721552 | 0.00064391 | NEDD8-MDP1 | 3.095255201 | 0.008151388 |
| ZNF763 | 2.569307917 | 4.8603E-05 | H2AC17 | 2.502406094 | 0.002464735 | ANKFN1 | 2.064911122 | 0.003188744 | AC010323.1 | 3.058772349 | 0.001972224 |
| CCL26 | 2.558633455 | 0.019010767 | AC004687.2 | 2.485066795 | 0.002569802 | NOXRED1 | 2.048177901 | 0.036022242 | TEX12 | 3.025936841 | 0.022546481 |
| PTPRR | 2.501524115 | 0.01218355 | AC017083.3 | 2.459033163 | 0.006747805 | AL139260.3 | 2.041538475 | 0.009292128 | FAP | 3.010705905 | 0.001609607 |
| AC087289.3 | 2.41876597 | 0.039801736 | EDN2 | 2.447179105 | 0.03886713 | POLR2J3 | 0.49860765 | 0.000212969 | ZNF460 | 3.001158116 | 2.94265E-23 |
| ANKRD37 | 2.365646007 | 0.007223553 | FGF21 | 2.395526643 | 0.024072238 | RASD2 | 0.49559872 | 0.013654175 | ADAMTS17 | 2.979058888 | 0.010509419 |
| KRT32 | 2.361950023 | 0.022512111 | VAX2 | 2.247922806 | 0.015099217 | DHRS9 | 0.493807921 | 0.007071909 | C17orf107 | 2.932636389 | 5.30568E-09 |
| TAS2R10 | 2.314218909 | 0.047017412 | ITGB6 | 2.231308768 | 0.008035168 | H2AC13 | 0.491765811 | 0.034829784 | TAS2R43 | 2.925352387 | 0.012692607 |
| AOC1 | 2.272143676 | 0.047870137 | H2AC20 | 2.230084119 | 0.009167373 | MAP1A | 0.48803064 | 2.96341E-14 | TMEM265 | 2.920185459 | 0.02507308 |
| IGFBP3 | 2.207258848 | 0.008901045 | ATP5MF-PTCD1 | 2.194838988 | 1.40867E-05 | WNT10B | 0.487994242 | 0.038488419 | EID3 | 2.913584265 | 0.000865438 |
| GGT5 | 2.194537562 | 0.03192455 | ANKRD23 | 2.189363022 | 0.0001832 | ZBED6 | 0.487817902 | 8.16156E-05 | CEP295NL | 2.904516059 | 0.015427147 |
| RAD51AP2 | 2.194306311 | 0.045391401 | PRB3 | 2.181969711 | 0.028349558 | ST20-MTHFS | 0.487056349 | 0.004202012 | CFP | 2.864723015 | 0.011798923 |
| ST6GALNAC3 | 2.17509716 | 0.047071083 | AC021087.5 | 2.158402466 | 6.67326E-07 | DLL1 | 0.486300867 | 0.000250136 | AC092718.8 | 2.822658091 | 0.000211974 |
| SOX30 | 2.112213451 | 0.038724667 | SLX1A | 2.110464234 | 0.022224323 | LAPTM5 | 0.485682656 | 0.006514184 | H3C1 | 2.820206575 | 0.025541432 |
| P4HA3 | 2.096317952 | 0.030778353 | MROH7 | 2.094745309 | 0.000284464 | ACE | 0.485467439 | 0.011567011 | H4C12 | 2.799397551 | 0.004420359 |
| DERPC | 2.09214918 | 0.024665037 | INHBE | 2.061011578 | 0.033495997 | H4C12 | 0.484543735 | 0.043621981 | H3C13 | 2.752806023 | 0.014233935 |
| NTF4 | 2.064080177 | 0.034235151 | INCA1 | 2.032444066 | 0.006728539 | AC099489.1 | 0.484025791 | 0.0004808 | GLOD5 | 2.74151482 | 0.013015765 |
| KLK11 | 2.05663824 | 0.035517261 | CNGA4 | 2.023642465 | 0.036992189 | HEPACAM2 | 0.48318346 | 4.36545E-08 | TEX29 | 2.727952758 | 0.022641408 |
| ABHD14A-ACY1 | 2.018499567 | 0.011032723 | MADCAM1 | 2.01085232 | 0.046341581 | PCDHAC2 | 0.482006422 | 0.003898849 | C20orf144 | 2.72329652 | 0.003797913 |
| CBLB | 0.498394082 | 0.035207253 | TMED6 | 0.499778798 | 0.042906477 | DKK1 | 0.481403182 | 3.83105E-09 | AC025283.2 | 2.722692961 | 0.004958328 |
| NFKBIA | 0.498217191 | 0.021045727 | NDUFC2-KCTD14 | 0.498607711 | 0.036716979 | TAS2R3 | 0.478027145 | 0.042576741 | DLG2 | 2.711262343 | 0.018190248 |
| SUSD3 | 0.495648077 | 0.047908869 | AL157935.2 | 0.489875717 | 0.00094118 | TENT5B | 0.47726845 | 0.018761422 | ATF3 | 2.655788679 | 9.5613E-220 |
| SPECC1L-ADORA2A | 0.49409262 | 0.021858456 | MEF2B | 0.487854439 | 0.000920646 | PADI3 | 0.475038087 | 0.013149245 | SLC22A1 | 2.579493538 | 0.020003017 |
| ST6GAL1 | 0.492225199 | 0.019118789 | CMPK2 | 0.477639629 | 0.003792155 | GDAP1 | 0.472403689 | 0.016605566 | CHRM4 | 2.550153691 | 0.034134521 |
| H2BU1 | 0.488827996 | 0.045683975 | CIDEB | 0.47359262 | 0.006363748 | CAMK1D | 0.47171383 | 0.024385985 | ALPI | 2.545388109 | 0.030820864 |
| TAP1 | 0.488803771 | 0.005617021 | AL022238.3 | 0.472834497 | 0.029828898 | LCN12 | 0.471631719 | 0.00027392 | H1-3 | 2.527142138 | 0.001645839 |
| HSPA1A | 0.487547268 | 0.023529898 | TUBAL3 | 0.470886952 | 0.033274524 | ZNF512B | 0.469256297 | 0.004180042 | AC018523.2 | 2.48485208 | 0.03983602 |
| GDPD3 | 0.484371903 | 0.046736141 | MAP1LC3B2 | 0.467723695 | 0.015646437 | SYNE1 | 0.469141882 | 4.85826E-05 | PLSCR2 | 2.482281498 | 0.002104768 |
| TP53INP1 | 0.484250099 | 0.007260769 | CFAP73 | 0.464037758 | 0.047049372 | C9orf24 | 0.468885446 | 0.031596898 | DIPK1B | 2.455075766 | 0.004492694 |
| PSMB10 | 0.483786436 | 0.049348314 | GNG3 | 0.460470557 | 0.037058426 | ABLIM2 | 0.468343251 | 0.0132948 | H4-16 | 2.444500368 | 8.94137E-07 |
| CDC42SE2 | 0.483600338 | 0.002754055 | FOXJ1 | 0.451209294 | 0.03987866 | LOXL2 | 0.468135371 | 1.36858E-05 | MROH7 | 2.439917187 | 0.018431263 |
| BEST1 | 0.483493276 | 0.038997096 | CYP4F8 | 0.448943339 | 0.007354699 | MUC5AC | 0.465080711 | 2.80112E-65 | RLN2 | 2.414491235 | 0.033174062 |
| PTK2B | 0.48181335 | 0.01285161 | A1BG | 0.440667082 | 0.012355917 | TAT | 0.46427201 | 0.018296979 | F10 | 2.397575016 | 0.045267961 |
| AC091057.6 | 0.479375629 | 0.014882325 | USHBP1 | 0.43908362 | 0.040974102 | CDC20 | 0.464098204 | 4.4789E-31 | RAB40AL | 2.376176169 | 0.016660945 |
| TNFRSF18 | 0.479331944 | 0.015038812 | GABRE | 0.433743102 | 0.01283111 | KIF20A | 0.460480998 | 3.5999E-19 | KLF2 | 2.362988411 | 8.78293E-14 |
| ADCY7 | 0.476293795 | 0.009522689 | ENTPD1 | 0.433379097 | 0.005152481 | DIPK1B | 0.458658165 | 0.011375285 | AC024592.3 | 2.324525556 | 0.022618547 |
| ARID5A | 0.47506435 | 0.021084092 | KRT86 | 0.43090419 | 0.008381835 | H2AC15 | 0.458433105 | 0.025398242 | ADHFE1 | 2.317003654 | 0.000486073 |
| PLCL2 | 0.473372293 | 0.02374272 | AL669918.1 | 0.429331013 | 0.014562192 | KLK14 | 0.456584288 | 0.021874877 | KIF19 | 2.304359932 | 0.018077482 |
| GPR155 | 0.472216547 | 0.020736652 | FOXC2 | 0.425963867 | 0.01935303 | SACS | 0.455780799 | 1.4879E-06 | CNTNAP1 | 2.285554893 | 0.012247715 |
| PLEKHF1 | 0.472039769 | 0.028076973 | DERPC | 0.424374238 | 0.001504336 | AL662884.4 | 0.454950113 | 0.040937957 | SPATA21 | 2.266096176 | 0.007642879 |
| CORO7 | 0.467511862 | 0.0446826 | TMEM253 | 0.419201812 | 0.048471871 | CNTNAP1 | 0.454108743 | 0.010210619 | ARMCX5-GPRASP2 | 2.253966739 | 0.020595676 |
| HECA | 0.463375652 | 0.008745184 | GNG2 | 0.418300906 | 0.000451772 | RAB3B | 0.453147802 | 1.53031E-06 | DLX1 | 2.252524455 | 0.031679292 |
| MDFIC | 0.463171578 | 0.012901891 | AC091167.2 | 0.411821187 | 0.021347805 | TTYH1 | 0.452516722 | 0.035907994 | TTYH2 | 2.239395669 | 0.020486099 |
| STK4 | 0.462055823 | 0.007254049 | CLTRN | 0.411670082 | 0.036055014 | FSD1 | 0.451871968 | 0.011845472 | WDR88 | 2.218743905 | 0.008448462 |
| JSRP1 | 0.46140264 | 0.025085866 | VAT1L | 0.410071991 | 0.01067849 | ABHD14A-ACY1 | 0.451642761 | 0.027239834 | RSPH4A | 2.203565821 | 0.018654762 |
| PSD4 | 0.461300004 | 0.011709144 | IFITM10 | 0.399255104 | 0.04153528 | HSPA6 | 0.450889616 | 0.018959843 | H2BC4 | 2.202427556 | 0.001040043 |
| KLRC4-KLRK1 | 0.459499212 | 0.037950342 | TMEM262 | 0.399077534 | 0.030942523 | AMOT | 0.449584993 | 0.002312903 | TMEM88 | 2.196805097 | 0.008648843 |
| COL5A3 | 0.459211456 | 0.04643117 | AC244197.3 | 0.392945971 | 0.01359115 | SPRR1B | 0.448726764 | 0.017064184 | KLRC4-KLRK1 | 2.152690837 | 0.019782632 |
| PCSK7 | 0.459026283 | 0.003930208 | KIAA1614 | 0.386930243 | 0.005109969 | AP003071.5 | 0.448383553 | 0.003845454 | ALDH1A2 | 2.145354344 | 0.02160134 |
| ANKAR | 0.458015542 | 0.03283477 | MEIOC | 0.382100655 | 3.06768E-05 | GPR161 | 0.448350801 | 0.010617883 | POLR2J2 | 2.134513635 | 0.002247985 |
| ERAP1 | 0.456399663 | 0.012982562 | KLF14 | 0.379221078 | 0.0036158 | HLTF | 0.446393912 | 0.002861989 | AL445685.3 | 2.123984624 | 0.000888775 |
| GPR15 | 0.456212225 | 0.017105405 | AC008763.2 | 0.368886332 | 0.017669135 | GEM | 0.443062649 | 0.000135633 | FAM47E-STBD1 | 2.12118856 | 0.006552065 |
| SDCBP2 | 0.454936351 | 0.039557836 | C4orf36 | 0.368207516 | 0.005494644 | TEX19 | 0.442671007 | 0.024566444 | CCDC102B | 2.120897072 | 0.025806225 |
| CCDC69 | 0.454240557 | 0.018653023 | FXYD7 | 0.367079749 | 0.01904484 | CACNA1H | 0.44254811 | 5.64827E-05 | SYNE1 | 2.103568302 | 0.006042745 |
| CFAP77 | 0.452326946 | 0.040356323 | FOXL2NB | 0.364537429 | 0.015774624 | DISP3 | 0.442214253 | 0.01677356 | AK8 | 2.090997844 | 0.004997256 |
| ERN1 | 0.451404195 | 0.003160983 | H4-16 | 0.36423449 | 0.002616575 | SPATA21 | 0.442195894 | 0.007218271 | TBC1D3K | 2.082383377 | 0.010166299 |
| BTG1 | 0.450697722 | 0.004315981 | ITGB3 | 0.359070668 | 0.003065222 | MRC2 | 0.442152796 | 0.021770675 | CHRNA3 | 2.067720887 | 0.021503774 |
| KCNAB2 | 0.450330509 | 0.010474431 | AL358113.1 | 0.34921779 | 0.01308907 | H2BC11 | 0.440579846 | 0.000348987 | DIRAS2 | 2.041242464 | 0.028689956 |
| TCF7 | 0.446588673 | 0.007420289 | NHLH1 | 0.345882443 | 0.002272785 | BEST2 | 0.436927517 | 0.010140024 | AC010522.1 | 2.02663609 | 0.010395722 |
| ARAP2 | 0.446518125 | 0.020448819 | CCDC38 | 0.338255457 | 0.028282715 | CEACAM5 | 0.436246628 | 6.94091E-08 | AC068896.1 | 2.019863826 | 0.013450605 |
| RHCE | 0.445922706 | 0.049546464 | TAS2R19 | 0.337549789 | 0.007586931 | ARHGAP11B | 0.435395587 | 1.22516E-13 | MUC16 | 2.011825433 | 0.000221469 |
| NOG | 0.445112578 | 0.033877133 | ALDH1L1 | 0.33159398 | 0.02201321 | UGT2B15 | 0.433445941 | 0.011026543 | SRCAP | 2.010035371 | 1.0196E-09 |
| SMAP1 | 0.443211074 | 0.043655185 | IRGM | 0.330072157 | 0.042329395 | B3GNT4 | 0.429280422 | 0.005033664 | ARPC4-TTLL3 | 2.004653207 | 0.021663862 |
| FAM220A | 0.442917602 | 0.019913434 | DCST2 | 0.328931676 | 0.000370141 | DACT1 | 0.428460136 | 0.029796121 | TMEM217 | 0.493571389 | 0.019695042 |
| FNBP1 | 0.44149185 | 0.006684689 | PRR4 | 0.313676033 | 0.006728867 | PLAGL1 | 0.427321218 | 0.001515902 | AL162231.1 | 0.49298048 | 0.047106169 |
| STX11 | 0.439798721 | 0.044166491 | MROH7-TTC4 | 0.305458348 | 6.86163E-08 | OLFM2 | 0.425661037 | 0.012772097 | OSCAR | 0.49215314 | 0.027536383 |
| ZBTB25 | 0.439470621 | 0.01314738 | GAPDHS | 0.280580304 | 0.018787669 | AC024592.3 | 0.425571438 | 0.016640191 | AC138894.1 | 0.48992265 | 0.035571697 |
| STAMBPL1 | 0.439076064 | 0.019619365 | ARMCX5-GPRASP2 | 0.276362936 | 1.77879E-07 | ADIRF | 0.424902409 | 0.0016202 | NINJ2 | 0.488657881 | 0.003303583 |
| BTN3A2 | 0.438784746 | 0.007666989 | CXCL17 | 0.2600578 | 0.014699688 | PRRT1B | 0.424417075 | 0.040349686 | MORN3 | 0.488204321 | 0.018429612 |
| EFEMP2 | 0.437012525 | 0.037942287 | FAM71F1 | 0.245678236 | 0.015446886 | QRICH2 | 0.423785657 | 0.006429746 | AC005520.1 | 0.486438995 | 0.005847836 |
| CPEB2 | 0.434444295 | 0.01858102 | AC092587.1 | 0.236583431 | 0.011157027 | FOXD4L3 | 0.421928997 | 0.022990929 | MTARC1 | 0.482799104 | 0.043398022 |
| SMAP2 | 0.433815633 | 0.011794877 | Z83844.2 | 0.227431453 | 2.56846E-07 | NPTXR | 0.420990075 | 0.000269846 | MSANTD1 | 0.482284482 | 0.025886239 |
| BMF | 0.431999813 | 0.035751202 | CCL26 | 0.222434472 | 0.000787334 | PRB3 | 0.420397915 | 0.02998371 | OGDHL | 0.471286807 | 0.000767954 |
| RAB27A | 0.430994691 | 0.01452473 | TEN1 | 0.218439336 | 5.23194E-08 | H2BC4 | 0.420278601 | 0.000101779 | FRZB | 0.47083043 | 0.027220025 |
| CISH | 0.430614365 | 0.036078828 | AD000671.2 | 0.212380201 | 0.029160825 | PCBP3 | 0.419275343 | 0.022100057 | C11orf1 | 0.46898009 | 0.01934641 |
| EOMES | 0.430337497 | 0.043064467 | SLC45A2 | 0.210628922 | 0.02600969 | TRIM17 | 0.413329737 | 0.005795852 | INCA1 | 0.448251651 | 0.020806175 |
| ANKRD23 | 0.43018945 | 0.00472643 | CGB5 | 0.202125118 | 0.006245466 | SNAI1 | 0.412846014 | 3.79919E-06 | AC025165.3 | 0.446428458 | 0.036118253 |
| BST2 | 0.428263858 | 0.021492472 | SLCO1B1 | 0.201687538 | 0.00081223 | UNC13A | 0.4095052 | 0.000178809 | AIF1L | 0.445168762 | 1.05382E-05 |
| NMRK1 | 0.427734517 | 0.015788459 | TVP23C-CDRT4 | 0.195069906 | 3.86922E-19 | MACROH2A2 | 0.40680804 | 0.032163318 | HIC1 | 0.444024781 | 8.57786E-05 |
| BTN3A3 | 0.426859507 | 0.013809828 | AC092647.5 | 0.179912074 | 0.011427621 | DOC2B | 0.406792674 | 0.000266136 | AC022384.1 | 0.436680316 | 0.029460402 |
| TBC1D2B | 0.423210835 | 0.009806965 | PRG2 | 0.175862633 | 0.002905573 | ATF3 | 0.405308136 | 6.1707E-137 | PFN4 | 0.43232142 | 0.027736696 |
| ATM | 0.422793492 | 0.018408186 | RUFY4 | 0.169443275 | 0.001188855 | CAMK2B | 0.404057717 | 0.035774104 | MUCL3 | 0.423883883 | 0.010457564 |
| B2M | 0.421730954 | 0.0009922 | SLC26A10 | 0.161647654 | 2.63749E-05 | KLRC4-KLRK1 | 0.400541854 | 0.037669221 | FMOD | 0.419971501 | 0.017768361 |
| H2AC17 | 0.421650367 | 0.029925169 | PKDCC | 0.160878196 | 0.000787677 | SPAG8 | 0.399546274 | 0.030680776 | AP000356.5 | 0.411691429 | 5.12676E-05 |
| CYTH1 | 0.421353419 | 0.002564411 | AC093884.1 | 0.160463918 | 0.001363859 | JSRP1 | 0.395649384 | 0.001265888 | PALM | 0.405197503 | 0.007851092 |
| SESN3 | 0.418539722 | 0.00698599 | FLACC1 | 0.153320535 | 0.000322605 | NEUROG3 | 0.389277606 | 0.000182763 | UTP14C | 0.402548934 | 0.026850099 |
| RUNX3 | 0.418499641 | 0.003899395 | AC104389.5 | 0.152698768 | 1.37841E-22 | ANGPTL2 | 0.385528457 | 0.024325868 | SPAAR | 0.386802017 | 0.022168812 |
| GHRHR | 0.418014082 | 0.017868406 | AC005833.1 | 0.150487738 | 0.007434036 | VWA5B2 | 0.38363974 | 0.009214805 | MMP13 | 0.385430145 | 0.007767927 |
| LRRC32 | 0.417598257 | 0.043547655 | H4C3 | 0.111048528 | 0.007091515 | H4-16 | 0.380681287 | 4.77446E-07 | VEGFD | 0.382483234 | 0.044362731 |
| VAX2 | 0.415831985 | 0.005384507 | AC068631.2 | 0.107209096 | 3.78063E-05 | COL6A1 | 0.378539428 | 1.55383E-08 | MAP6D1 | 0.381625687 | 0.010039577 |
| PPM1K | 0.41567971 | 0.022217304 | SMCP | 0.072941056 | 0.000217746 | AC092718.8 | 0.376637104 | 0.007808568 | ATP5F1A | 0.380170399 | 7.87397E-07 |
| CAMK4 | 0.415434771 | 0.015506362 | ZNF816-ZNF321P | 0.063746645 | 1.89757E-06 | C1S | 0.372554374 | 8.31168E-05 | AC004080.3 | 0.367055213 | 0.012916857 |
| IL21R | 0.414824135 | 0.007669018 | WFDC10B | 0.061727442 | 0.00039344 | SCG2 | 0.371583558 | 0.00118904 | CENPS-CORT | 0.364831361 | 0.01486215 |
| ADCY4 | 0.413500462 | 0.03470676 | COLEC11 | 0.059937665 | 9.40459E-08 | OLFML3 | 0.371135661 | 0.02244745 | SULT1C2 | 0.356018601 | 0.012981911 |
| FOXP1 | 0.413485146 | 0.019035321 | HLA-DRB5 | 0.049734418 | 1.173E-07 | SSUH2 | 0.371100315 | 0.001730834 | MT1F | 0.348089701 | 0.008580683 |
| FAM78A | 0.407742987 | 0.027527403 | HSD17B3 | 0.049677469 | 3.12542E-07 | CA8 | 0.371081931 | 0.000147246 | NPHP3-ACAD11 | 0.347626964 | 0.010134111 |
| TRABD2A | 0.403096731 | 0.013002779 | AC242842.3 | 0.048645872 | 2.34089E-23 | TOX2 | 0.370861454 | 0.003932106 | PRF1 | 0.345108423 | 0.001353935 |
| FAM174B | 0.400569033 | 0.005879501 | PINX1 | 0.03966381 | 2.30926E-11 | GUCA2B | 0.364718421 | 0.007149799 | SLC25A41 | 0.342076248 | 0.017811922 |
| NCALD | 0.400469645 | 0.026214058 | AC018523.2 | 0.007657583 | 2.57965E-25 | ABCC2 | 0.364435994 | 1.10661E-09 | UTS2B | 0.341581917 | 0.027961608 |
| RNF213 | 0.400163958 | 0.010009844 |  |  |  | NLRP2 | 0.363479661 | 1.65275E-05 | COL9A2 | 0.340651072 | 0.004155976 |
| EIF4E1B | 0.399572241 | 0.01683315 |  |  |  | PRPH | 0.362402348 | 0.005937652 | FAM177B | 0.338007157 | 0.022048913 |
| AP001931.2 | 0.398488282 | 8.16445E-05 |  |  |  | DNAJB5 | 0.361897796 | 0.006307678 | PMF1-BGLAP | 0.337388442 | 0.000432755 |
| DPYD | 0.396658079 | 0.044259527 |  |  |  | PMP22 | 0.361018432 | 0.027019452 | H3C2 | 0.332765424 | 0.014620624 |
| SEMA4D | 0.396488529 | 0.00476346 |  |  |  | GLIPR2 | 0.360558938 | 0.025165641 | GUCA1B | 0.32148328 | 0.00066706 |
| PHF11 | 0.39566783 | 0.029695666 |  |  |  | ACHE | 0.358827128 | 0.033718804 | AL031315.1 | 0.310925943 | 0.022492659 |
| ZNF550 | 0.39535624 | 0.018690756 |  |  |  | AC018523.2 | 0.357910839 | 0.006842631 | AC004997.1 | 0.304703452 | 0.000129418 |
| HLA-A | 0.394313015 | 0.001067941 |  |  |  | MSX1 | 0.356326713 | 0.000165045 | ASGR1 | 0.304441023 | 0.004094612 |
| CD82 | 0.393145105 | 0.00887945 |  |  |  | H2BC15 | 0.356263585 | 1.09457E-05 | CCL20 | 0.29778203 | 0.005590538 |
| SYNE1 | 0.39259081 | 0.014578085 |  |  |  | H2AC17 | 0.354383694 | 0.043484468 | FSD1 | 0.297335379 | 0.006200009 |
| PRKCH | 0.392337732 | 0.005288978 |  |  |  | PLAU | 0.353489206 | 8.35602E-05 | SLC4A9 | 0.297207208 | 0.007967779 |
| MLLT11 | 0.390976046 | 0.006720281 |  |  |  | SRPX | 0.353462197 | 0.003623665 | GRAP | 0.297036199 | 0.020910144 |
| PREX1 | 0.390493721 | 0.008202019 |  |  |  | MYL9 | 0.347663948 | 0.000598636 | ADA | 0.295383342 | 0.008585735 |
| NRN1 | 0.389928027 | 0.047926574 |  |  |  | SHANK3 | 0.344087888 | 4.50093E-05 | AC069288.1 | 0.288572859 | 0.001548109 |
| GPRASP1 | 0.389717324 | 0.046450188 |  |  |  | RTBDN | 0.343593144 | 0.01810488 | FGF11 | 0.287780302 | 0.001341636 |
| CD274 | 0.389264978 | 0.044656315 |  |  |  | CXCL8 | 0.342728513 | 2.91498E-10 | CELF6 | 0.277956162 | 0.000111792 |
| CARD8 | 0.387430936 | 0.002407284 |  |  |  | GFY | 0.342546464 | 0.001740444 | CD164L2 | 0.27170947 | 0.020232737 |
| TENT5C | 0.387407229 | 0.007807587 |  |  |  | DLEC1 | 0.339280413 | 0.000223423 | HEY1 | 0.268556837 | 0.004535528 |
| CLK1 | 0.386833427 | 0.003431439 |  |  |  | EVL | 0.33652928 | 0.001057987 | TBC1D3B | 0.266262367 | 0.023517418 |
| MCTP2 | 0.385993238 | 0.028809453 |  |  |  | SPON1 | 0.334439133 | 0.008423021 | AC091057.6 | 0.265068052 | 4.96416E-11 |
| PRDM1 | 0.385482018 | 0.017660136 |  |  |  | AL669918.1 | 0.334430036 | 0.013597217 | ENTPD1 | 0.259967095 | 0.000276695 |
| TMEM269 | 0.384229283 | 0.028755542 |  |  |  | RPA4 | 0.331625221 | 0.015501956 | AP003419.1 | 0.253188694 | 0.023770408 |
| HLA-E | 0.384177527 | 0.001257175 |  |  |  | LRRC53 | 0.328922851 | 0.000473782 | ANGPTL3 | 0.252156453 | 0.012827744 |
| PDE7A | 0.382472565 | 0.011576409 |  |  |  | SOCS3 | 0.328826858 | 0.000798153 | AL358113.1 | 0.242255457 | 0.00475975 |
| AC244197.3 | 0.37910426 | 0.036393788 |  |  |  | NCF2 | 0.328619858 | 6.84232E-07 | AC018512.1 | 0.241907459 | 2.58388E-07 |
| DCHS1 | 0.37773817 | 0.01419282 |  |  |  | RTP3 | 0.327999721 | 0.007709353 | ATP1A4 | 0.2361603 | 0.005088573 |
| AC008763.2 | 0.377226245 | 0.008515436 |  |  |  | ALDH1A2 | 0.327286996 | 0.000235887 | PPAN-P2RY11 | 0.220139617 | 0.023683859 |
| RNF19A | 0.374238249 | 0.006144572 |  |  |  | DLG2 | 0.324452733 | 0.011559908 | AP001931.2 | 0.217342021 | 1.27044E-57 |
| AC068775.1 | 0.369008735 | 0.005898044 |  |  |  | LY6G6C | 0.324188348 | 0.019745475 | KLRK1 | 0.211497191 | 0.00333167 |
| ARL4C | 0.36757239 | 0.006576659 |  |  |  | PHOSPHO1 | 0.323521113 | 0.000913678 | MAGEB2 | 0.207321362 | 0.003296792 |
| KCTD7 | 0.363564282 | 0.005808595 |  |  |  | NPW | 0.322951418 | 0.006715766 | C16orf71 | 0.200198385 | 0.000469304 |
| LYST | 0.363276036 | 0.005096267 |  |  |  | TSNAX-DISC1 | 0.322949151 | 9.06843E-05 | CORO6 | 0.188586666 | 1.47411E-05 |
| CCDC88C | 0.362661994 | 0.007605095 |  |  |  | H4C15 | 0.3224755 | 0.000425088 | DUS4L-BCAP29 | 0.18442789 | 3.1649E-25 |
| PRDM8 | 0.361895797 | 0.023701479 |  |  |  | MUC2 | 0.322053646 | 1.73235E-48 | AD000671.2 | 0.181548223 | 0.002221206 |
| KAT2B | 0.361509564 | 0.013029157 |  |  |  | HSPB8 | 0.321774066 | 0.016586251 | CYP2C9 | 0.179223581 | 0.002784204 |
| SEPTIN6 | 0.361234989 | 0.003511375 |  |  |  | AC004687.2 | 0.319346464 | 4.56073E-08 | CKMT2 | 0.178470559 | 0.005432417 |
| STK17B | 0.360591967 | 0.003053674 |  |  |  | C20orf144 | 0.319163794 | 0.000448961 | CITED1 | 0.155369392 | 0.000136245 |
| KLRC1 | 0.360468158 | 0.003581519 |  |  |  | FCGBP | 0.317639506 | 6.6187E-143 | SERF1A | 0.110861013 | 0.000505819 |
| ABCA1 | 0.358901741 | 0.011252504 |  |  |  | RFLNB | 0.315082778 | 0.000610955 | ITIH4 | 0.110656384 | 0.009166921 |
| TRPV2 | 0.356840825 | 0.015013802 |  |  |  | CABYR | 0.314769982 | 0.000991861 | CORO7-PAM16 | 0.084007905 | 3.16119E-06 |
| SMIM27 | 0.35655815 | 0.045675764 |  |  |  | AL662899.2 | 0.313309227 | 0.001281435 | AS3MT | 0.083572211 | 2.73475E-08 |
| EXO5 | 0.355434562 | 0.033888261 |  |  |  | ID3 | 0.31239376 | 5.23389E-05 | HIPK4 | 0.083118265 | 3.0427E-08 |
| C8orf44-SGK3 | 0.354880169 | 0.008734796 |  |  |  | FSCN1 | 0.31206269 | 2.67157E-07 | AP002990.1 | 0.082747694 | 1.1252E-144 |
| AMIGO3 | 0.353552653 | 0.013018941 |  |  |  | PSTPIP2 | 0.311662761 | 3.98871E-05 | AC008878.1 | 0.072070905 | 0.000163175 |
| LPIN2 | 0.353244769 | 0.00297469 |  |  |  | IFNB1 | 0.311084732 | 0.003074153 | AP001273.2 | 0.069227863 | 0.000126525 |
| INHBE | 0.35057211 | 0.014898885 |  |  |  | RBPMS2 | 0.307977265 | 8.26056E-05 | IFNL2 | 0.066406354 | 1.02194E-05 |
| HLA-B | 0.350536591 | 0.000901006 |  |  |  | GAL3ST2 | 0.306157711 | 0.037255604 | CLPSL1 | 0.050670754 | 2.7686E-05 |
| BTN3A1 | 0.349490309 | 0.004497582 |  |  |  | GFAP | 0.304585049 | 0.003471499 | AC009163.3 | 0.045992672 | 1.10423E-12 |
| CARD9 | 0.349066031 | 0.003042563 |  |  |  | TEX29 | 0.302365116 | 0.004546211 | BEST1 | 0.032511465 | 0 |
| FAM50B | 0.347699998 | 0.045158001 |  |  |  | UTP14C | 0.301809192 | 8.17464E-10 | CATSPERZ | 0.027599426 | 2.72012E-12 |
| AD000671.2 | 0.347327701 | 0.030713754 |  |  |  | BCAN | 0.301778594 | 0.000917977 | TFF1 | 0.015754606 | 5.49337E-09 |
| ATXN1 | 0.346573896 | 0.001832674 |  |  |  | TREX1 | 0.30109514 | 7.64942E-07 | HSPE1-MOB4 | 0.015043374 | 2.62192E-10 |
| CSF1 | 0.346422081 | 0.024911165 |  |  |  | PLB1 | 0.297936425 | 0.000145812 |  |  |  |
| ADRB2 | 0.346153695 | 0.016136136 |  |  |  | ENTPD1 | 0.294854133 | 0.007233864 |  |  |  |
| CFLAR | 0.345341376 | 0.008751125 |  |  |  | ANO3 | 0.293035263 | 0.015454332 |  |  |  |
| FLT3LG | 0.345170066 | 0.003346985 |  |  |  | CALD1 | 0.292459326 | 2.21555E-06 |  |  |  |
| ANTXR2 | 0.344383923 | 0.007997908 |  |  |  | FGFBP1 | 0.291998957 | 0.003879609 |  |  |  |
| TMSB4X | 0.342631715 | 0.002025179 |  |  |  | LY75-CD302 | 0.290500432 | 1.1463E-07 |  |  |  |
| FYN | 0.341398559 | 0.007704733 |  |  |  | SLC1A3 | 0.286109514 | 4.97288E-05 |  |  |  |
| GK | 0.3409708 | 0.014885026 |  |  |  | SLC10A4 | 0.284493565 | 0.000425762 |  |  |  |
| BICDL1 | 0.340783591 | 0.004284308 |  |  |  | CD109 | 0.283497106 | 3.89557E-10 |  |  |  |
| REM2 | 0.340483291 | 0.025626114 |  |  |  | PDE4C | 0.280443701 | 3.79375E-11 |  |  |  |
| DHX58 | 0.338987638 | 0.013005224 |  |  |  | AOAH | 0.279854163 | 0.00579825 |  |  |  |
| PPP1R3E | 0.335417596 | 0.005369969 |  |  |  | PDLIM4 | 0.273781827 | 0.000687208 |  |  |  |
| MPZL3 | 0.334276914 | 0.002751915 |  |  |  | TENM1 | 0.273235225 | 3.25925E-11 |  |  |  |
| KLRC4 | 0.332850929 | 0.021959329 |  |  |  | GSDME | 0.27290126 | 6.67039E-06 |  |  |  |
| SLC16A4 | 0.332569503 | 0.018323069 |  |  |  | TPTEP2-CSNK1E | 0.269407895 | 0.000261396 |  |  |  |
| CYLD | 0.331046759 | 0.000753032 |  |  |  | SLC43A3 | 0.266665276 | 0.000283108 |  |  |  |
| DGKE | 0.330665935 | 0.004612539 |  |  |  | PHLDB2 | 0.264993049 | 6.41932E-12 |  |  |  |
| PIM2 | 0.326608723 | 0.002566571 |  |  |  | OSMR | 0.263456987 | 2.52955E-07 |  |  |  |
| GOLGA8R | 0.322849143 | 0.01864602 |  |  |  | FER1L6 | 0.259366303 | 5.19314E-07 |  |  |  |
| PIK3CD | 0.32144489 | 0.001904809 |  |  |  | ATP8B2 | 0.250531097 | 2.30707E-06 |  |  |  |
| CASS4 | 0.320261589 | 0.019329555 |  |  |  | GFPT2 | 0.245947497 | 3.58218E-05 |  |  |  |
| ASDURF | 0.317223645 | 0.029556901 |  |  |  | ATOH7 | 0.244940889 | 0.000487239 |  |  |  |
| ERFL | 0.316058314 | 0.023750819 |  |  |  | GALNT18 | 0.244857892 | 0.000127634 |  |  |  |
| AC073111.4 | 0.315195983 | 0.013210218 |  |  |  | CPE | 0.244600296 | 1.25765E-05 |  |  |  |
| CCDC65 | 0.314973473 | 0.034864876 |  |  |  | H3C13 | 0.244542935 | 0.000469026 |  |  |  |
| PTGER4 | 0.314648111 | 0.002481001 |  |  |  | LIPK | 0.244247206 | 0.000504506 |  |  |  |
| SOX7 | 0.314590195 | 0.002793994 |  |  |  | CALB1 | 0.242389772 | 0.000747497 |  |  |  |
| IL23A | 0.313966875 | 0.001756603 |  |  |  | SPDYE16 | 0.242157942 | 0.000758208 |  |  |  |
| MGAT4A | 0.313404105 | 0.00403052 |  |  |  | ELOVL5 | 0.241079724 | 1.39824E-07 |  |  |  |
| PCP2 | 0.312916419 | 0.021518652 |  |  |  | CHRM4 | 0.240440304 | 0.000392705 |  |  |  |
| ATP5MF-PTCD1 | 0.312689465 | 6.76365E-05 |  |  |  | ANKLE1 | 0.240073186 | 2.03069E-05 |  |  |  |
| ZNF615 | 0.312005247 | 0.03330843 |  |  |  | CFAP20DC | 0.235878536 | 8.44846E-06 |  |  |  |
| RGCC | 0.309478833 | 0.012617223 |  |  |  | CFAP161 | 0.23574697 | 0.0026269 |  |  |  |
| GP1BB | 0.308014254 | 0.015782355 |  |  |  | TOMM6 | 0.230411466 | 0.007677358 |  |  |  |
| AKNA | 0.307867244 | 0.000954111 |  |  |  | TGFBR3L | 0.219769929 | 0.003346577 |  |  |  |
| IL18R1 | 0.307854131 | 0.004399174 |  |  |  | SOCS2 | 0.21931994 | 0.000221137 |  |  |  |
| RNASET2 | 0.306236945 | 0.018951084 |  |  |  | NGFR | 0.218340457 | 1.23395E-06 |  |  |  |
| IFITM1 | 0.30597597 | 0.008819848 |  |  |  | PTPRU | 0.217845217 | 8.68173E-08 |  |  |  |
| HLA-F | 0.305698163 | 0.00126455 |  |  |  | RNF112 | 0.217672231 | 0.0024598 |  |  |  |
| ARHGAP45 | 0.30469057 | 0.000701655 |  |  |  | PCDH7 | 0.215097265 | 2.45583E-10 |  |  |  |
| PDZD4 | 0.299029956 | 0.007106266 |  |  |  | ZNF518B | 0.212859761 | 3.82367E-07 |  |  |  |
| CACNA1I | 0.291828977 | 0.007781817 |  |  |  | COL12A1 | 0.211756864 | 7.41183E-11 |  |  |  |
| BGLAP | 0.290418071 | 0.026360796 |  |  |  | PI3 | 0.211165337 | 0.005601539 |  |  |  |
| ENG | 0.290013302 | 0.014950137 |  |  |  | RASGEF1A | 0.210791445 | 4.95392E-05 |  |  |  |
| AL096711.2 | 0.288621607 | 0.009297678 |  |  |  | AC091167.2 | 0.210380538 | 0.013148349 |  |  |  |
| IQGAP2 | 0.288070715 | 0.009691285 |  |  |  | TNF | 0.209288441 | 0.000164465 |  |  |  |
| YPEL4 | 0.287671196 | 0.048322825 |  |  |  | ANTXR1 | 0.208719112 | 3.63655E-09 |  |  |  |
| SSX1 | 0.287337662 | 0.025933194 |  |  |  | PTPRG | 0.207590692 | 4.04134E-13 |  |  |  |
| ITPKB | 0.286364035 | 0.001347806 |  |  |  | BNIP3 | 0.206568834 | 1.9146E-06 |  |  |  |
| ALG1L2 | 0.286070783 | 0.010251209 |  |  |  | AKAP12 | 0.202234992 | 2.28251E-79 |  |  |  |
| IZUMO4 | 0.285301569 | 0.005364734 |  |  |  | WFDC3 | 0.202178603 | 0.00077071 |  |  |  |
| DAPP1 | 0.284350979 | 0.013885248 |  |  |  | FAM47E-STBD1 | 0.198792449 | 6.24224E-07 |  |  |  |
| UTS2 | 0.282448578 | 0.019566571 |  |  |  | IL1A | 0.190799523 | 0.0001761 |  |  |  |
| H4C11 | 0.281746247 | 0.048200359 |  |  |  | LY6D | 0.18866213 | 0.001381925 |  |  |  |
| AKAP5 | 0.281726616 | 0.008659402 |  |  |  | FGFR1 | 0.188226762 | 3.33281E-08 |  |  |  |
| CALCB | 0.281131792 | 0.036681753 |  |  |  | ACAP1 | 0.185723249 | 1.50901E-06 |  |  |  |
| GLCCI1 | 0.27989623 | 0.005158312 |  |  |  | AC093512.2 | 0.182607158 | 0.000291652 |  |  |  |
| AC013394.1 | 0.276658464 | 0.008692069 |  |  |  | CHST11 | 0.179530783 | 0.001584154 |  |  |  |
| CLIC5 | 0.27115347 | 0.014980784 |  |  |  | MAGEB2 | 0.177054524 | 7.99874E-10 |  |  |  |
| ITGA1 | 0.270442079 | 0.004167034 |  |  |  | DDIT4L | 0.17551575 | 7.74481E-06 |  |  |  |
| PLCB2 | 0.2702927 | 0.001039287 |  |  |  | MAP1B | 0.174475878 | 1.60373E-09 |  |  |  |
| TOGARAM2 | 0.266461762 | 0.030046769 |  |  |  | B4GALNT4 | 0.174124026 | 1.02933E-05 |  |  |  |
| HLA-DRA | 0.2635779 | 0.002411677 |  |  |  | STOM | 0.173097478 | 0.001149541 |  |  |  |
| ABCG1 | 0.263223857 | 0.015752927 |  |  |  | TUBB4A | 0.172716779 | 7.13168E-09 |  |  |  |
| PRKCQ | 0.262499834 | 0.00477256 |  |  |  | AC010323.1 | 0.171925585 | 4.95857E-05 |  |  |  |
| TSC22D3 | 0.261636686 | 0.000669372 |  |  |  | CAPNS2 | 0.169706324 | 0.00046913 |  |  |  |
| LIMD2 | 0.258548688 | 0.006311201 |  |  |  | KCNG1 | 0.168825253 | 7.01902E-10 |  |  |  |
| RUNX2 | 0.257441751 | 0.002310377 |  |  |  | AC025283.2 | 0.167571075 | 2.32764E-07 |  |  |  |
| RASSF5 | 0.255580029 | 0.001171454 |  |  |  | GAL | 0.163995997 | 0.000458923 |  |  |  |
| SLC7A10 | 0.255045992 | 0.004259721 |  |  |  | ONECUT3 | 0.161868287 | 6.72215E-11 |  |  |  |
| C1orf56 | 0.252496301 | 0.003788801 |  |  |  | FHL1 | 0.160970686 | 7.36689E-07 |  |  |  |
| GPC5 | 0.251642663 | 0.014289629 |  |  |  | AKR1B1 | 0.160024386 | 1.83727E-12 |  |  |  |
| ACY3 | 0.251500785 | 0.02842381 |  |  |  | MMP1 | 0.157779379 | 4.95276E-06 |  |  |  |
| MFNG | 0.247383243 | 0.003245111 |  |  |  | MATK | 0.157074439 | 3.24142E-06 |  |  |  |
| PLCH2 | 0.24737883 | 0.005334492 |  |  |  | LGALS7 | 0.156855334 | 0.000634159 |  |  |  |
| YPEL3 | 0.245409596 | 0.001629031 |  |  |  | SLC22A31 | 0.156026177 | 1.43211E-06 |  |  |  |
| C5AR2 | 0.244155899 | 0.024071698 |  |  |  | FAP | 0.153569539 | 0.000264629 |  |  |  |
| POU2F2 | 0.240850466 | 0.00305129 |  |  |  | RTL8C | 0.153158881 | 5.58372E-05 |  |  |  |
| RCBTB2 | 0.240094277 | 0.017142069 |  |  |  | KRTAP2-3 | 0.152278914 | 0.000589645 |  |  |  |
| SATB1 | 0.240071202 | 0.0016755 |  |  |  | KCNH3 | 0.149384145 | 3.93797E-09 |  |  |  |
| AC010325.1 | 0.239801402 | 0.047338888 |  |  |  | COL6A2 | 0.148820257 | 1.68844E-14 |  |  |  |
| GFI1 | 0.239052832 | 0.000589215 |  |  |  | MYH15 | 0.140799419 | 4.18046E-13 |  |  |  |
| SH2D3C | 0.23188807 | 0.003787221 |  |  |  | PTPRS | 0.139519958 | 4.6433E-11 |  |  |  |
| LDLRAD4 | 0.23089127 | 0.015394498 |  |  |  | AMOTL1 | 0.138701986 | 1.64338E-08 |  |  |  |
| SLX1A | 0.230864417 | 0.000309401 |  |  |  | GTSF1 | 0.135916093 | 0.005771239 |  |  |  |
| NLRC5 | 0.229500473 | 0.000913192 |  |  |  | BEX2 | 0.133666121 | 0.006231443 |  |  |  |
| SKAP1 | 0.229275757 | 0.002319215 |  |  |  | SYT1 | 0.133486766 | 3.00276E-09 |  |  |  |
| PRSS57 | 0.228825028 | 0.02733193 |  |  |  | CORT | 0.132106879 | 0.000119879 |  |  |  |
| ANGPTL6 | 0.227689966 | 0.00441558 |  |  |  | CHST15 | 0.129082469 | 4.89448E-06 |  |  |  |
| SPON2 | 0.2273276 | 0.008913252 |  |  |  | HS6ST2 | 0.125435659 | 2.03145E-10 |  |  |  |
| AC069288.1 | 0.22721512 | 0.000199526 |  |  |  | ARL14EPL | 0.125349782 | 0.001268468 |  |  |  |
| ZEB1 | 0.225043555 | 0.001723012 |  |  |  | COL4A5 | 0.124855456 | 1.4276E-05 |  |  |  |
| ATP6V1G2-DDX39B | 0.222667212 | 0.005200469 |  |  |  | C6orf15 | 0.119083963 | 3.99531E-07 |  |  |  |
| RFTN1 | 0.222626947 | 0.000875913 |  |  |  | TSPAN7 | 0.117930336 | 6.58283E-07 |  |  |  |
| RENBP | 0.218132842 | 0.000203908 |  |  |  | FBXO17 | 0.11722408 | 1.35971E-07 |  |  |  |
| HLA-DQB1 | 0.217469555 | 0.009822868 |  |  |  | CERS4 | 0.116988668 | 0.000773114 |  |  |  |
| STAT4 | 0.215331083 | 0.002508556 |  |  |  | AC027796.3 | 0.115971543 | 0.002415532 |  |  |  |
| PDE3B | 0.215251724 | 0.00099748 |  |  |  | CD22 | 0.112059992 | 3.2604E-08 |  |  |  |
| NLRP1 | 0.214865058 | 0.000698195 |  |  |  | HBG2 | 0.111006066 | 9.55437E-05 |  |  |  |
| GATA3 | 0.214699618 | 0.001059262 |  |  |  | SPDYE21 | 0.109829199 | 3.93134E-05 |  |  |  |
| CORO1A | 0.211509355 | 0.000146205 |  |  |  | ITGA5 | 0.109072065 | 3.02389E-06 |  |  |  |
| GAB3 | 0.208483661 | 0.004126621 |  |  |  | KBTBD11-OT1 | 0.098466549 | 1.88824E-06 |  |  |  |
| ROPN1L | 0.205413251 | 0.001047007 |  |  |  | FAM25A | 0.09215956 | 1.40816E-05 |  |  |  |
| AC007192.1 | 0.203639923 | 1.63173E-08 |  |  |  | HBE1 | 0.089126114 | 6.58832E-11 |  |  |  |
| JAK3 | 0.200448652 | 0.000346345 |  |  |  | PLAT | 0.073682441 | 1.43445E-11 |  |  |  |
| CYTH4 | 0.200107096 | 0.001583739 |  |  |  | H3C3 | 0.073393156 | 5.42526E-05 |  |  |  |
| TMC8 | 0.20002856 | 0.000310445 |  |  |  | ARL2-SNX15 | 0.070006196 | 9.69544E-11 |  |  |  |
| SH2D2A | 0.197496317 | 7.76217E-05 |  |  |  | PGAP4 | 0.069926876 | 2.12748E-06 |  |  |  |
| DENND1C | 0.197306746 | 0.001113196 |  |  |  | DUSP9 | 0.067945861 | 1.03204E-10 |  |  |  |
| AC135050.2 | 0.196274391 | 0.000364741 |  |  |  | OR51B5 | 0.061743692 | 1.37459E-15 |  |  |  |
| H3C1 | 0.19566362 | 0.0072007 |  |  |  | CD40 | 0.060180059 | 2.4841E-09 |  |  |  |
| NR4A3 | 0.194749549 | 0.001004683 |  |  |  | PRAME | 0.056476435 | 1.2364E-13 |  |  |  |
| APOBEC3G | 0.194632571 | 0.002529589 |  |  |  | SLCO1B3-SLCO1B7 | 0.055311523 | 8.48821E-06 |  |  |  |
| TEX13B | 0.193641189 | 0.004919319 |  |  |  | TEX46 | 0.053830874 | 7.50183E-05 |  |  |  |
| TEKT5 | 0.193494166 | 0.005274176 |  |  |  | AC004922.1 | 0.053811623 | 7.12895E-14 |  |  |  |
| STPG2 | 0.189746695 | 0.005158055 |  |  |  | LPAR3 | 0.048721072 | 1.68667E-17 |  |  |  |
| SYT11 | 0.188633236 | 0.006121718 |  |  |  | BEX3 | 0.047244582 | 3.92713E-11 |  |  |  |
| C1orf162 | 0.188341982 | 0.001466898 |  |  |  | H3C12 | 0.040302023 | 9.41033E-06 |  |  |  |
| MMP25 | 0.187868599 | 0.002067571 |  |  |  | KCNQ2 | 0.032013442 | 6.67943E-08 |  |  |  |
| AKT3 | 0.186320626 | 0.002035638 |  |  |  | OR51B4 | 0.030242957 | 1.02155E-09 |  |  |  |
| HSPE1-MOB4 | 0.185990928 | 0.001494109 |  |  |  |  |  |  |  |  |  |
| AC005324.3 | 0.184034227 | 2.47327E-07 |  |  |  |  |  |  |  |  |  |
| CX3CR1 | 0.183368476 | 0.002034227 |  |  |  |  |  |  |  |  |  |
| TMEM256-PLSCR3 | 0.182927196 | 7.04196E-12 |  |  |  |  |  |  |  |  |  |
| ANKRD44 | 0.182245858 | 0.000947747 |  |  |  |  |  |  |  |  |  |
| TRANK1 | 0.181845824 | 0.001199068 |  |  |  |  |  |  |  |  |  |
| KLRG1 | 0.180582216 | 0.000915746 |  |  |  |  |  |  |  |  |  |
| ITGAX | 0.177849675 | 0.001621517 |  |  |  |  |  |  |  |  |  |
| NEDD9 | 0.176131425 | 0.002763796 |  |  |  |  |  |  |  |  |  |
| BCL2 | 0.175641473 | 0.000396106 |  |  |  |  |  |  |  |  |  |
| HLA-DPA1 | 0.175449053 | 0.001340109 |  |  |  |  |  |  |  |  |  |
| SPATC1L | 0.17477418 | 0.003141152 |  |  |  |  |  |  |  |  |  |
| RASGEF1B | 0.173926024 | 0.002542086 |  |  |  |  |  |  |  |  |  |
| AL160269.1 | 0.172807863 | 8.35461E-07 |  |  |  |  |  |  |  |  |  |
| PLXNC1 | 0.171743986 | 0.000461705 |  |  |  |  |  |  |  |  |  |
| CCR3 | 0.171726992 | 0.00482796 |  |  |  |  |  |  |  |  |  |
| GLIPR1 | 0.171393158 | 0.000302763 |  |  |  |  |  |  |  |  |  |
| NRN1L | 0.171265833 | 0.026486765 |  |  |  |  |  |  |  |  |  |
| TCAF2 | 0.170128712 | 0.001259148 |  |  |  |  |  |  |  |  |  |
| HLA-DRB1 | 0.169223 | 0.001102277 |  |  |  |  |  |  |  |  |  |
| SELL | 0.166840474 | 0.000511682 |  |  |  |  |  |  |  |  |  |
| SAMD3 | 0.165306131 | 0.000620487 |  |  |  |  |  |  |  |  |  |
| SP140 | 0.164628004 | 0.000975992 |  |  |  |  |  |  |  |  |  |
| PIK3R5 | 0.164626475 | 0.000248162 |  |  |  |  |  |  |  |  |  |
| MEI1 | 0.161802513 | 0.011881449 |  |  |  |  |  |  |  |  |  |
| STAT5B | 0.16162042 | 0.000273311 |  |  |  |  |  |  |  |  |  |
| PAG1 | 0.161477227 | 0.000329923 |  |  |  |  |  |  |  |  |  |
| CD79B | 0.16039701 | 0.001375759 |  |  |  |  |  |  |  |  |  |
| FGD3 | 0.159395744 | 0.001169566 |  |  |  |  |  |  |  |  |  |
| RNF157 | 0.158811754 | 0.002033694 |  |  |  |  |  |  |  |  |  |
| GALNT8 | 0.155503349 | 0.006343839 |  |  |  |  |  |  |  |  |  |
| GBP3 | 0.154516816 | 0.003108699 |  |  |  |  |  |  |  |  |  |
| ADAM8 | 0.153714964 | 0.000247889 |  |  |  |  |  |  |  |  |  |
| ERAP2 | 0.152197795 | 0.000631496 |  |  |  |  |  |  |  |  |  |
| ARHGEF6 | 0.150505072 | 0.000237768 |  |  |  |  |  |  |  |  |  |
| ITGAM | 0.150323798 | 0.007207355 |  |  |  |  |  |  |  |  |  |
| VAMP5 | 0.150286637 | 0.001509301 |  |  |  |  |  |  |  |  |  |
| BEX4 | 0.148444454 | 0.005837411 |  |  |  |  |  |  |  |  |  |
| IKZF2 | 0.14807786 | 0.000878466 |  |  |  |  |  |  |  |  |  |
| KLRK1 | 0.147902314 | 0.000914912 |  |  |  |  |  |  |  |  |  |
| N4BP2L1 | 0.145997685 | 0.001191984 |  |  |  |  |  |  |  |  |  |
| CORO7-PAM16 | 0.144885218 | 5.52388E-05 |  |  |  |  |  |  |  |  |  |
| JAKMIP3 | 0.143927595 | 0.00327148 |  |  |  |  |  |  |  |  |  |
| IGFLR1 | 0.142135946 | 3.12104E-05 |  |  |  |  |  |  |  |  |  |
| AC055839.2 | 0.141689819 | 0.000377513 |  |  |  |  |  |  |  |  |  |
| INPP5D | 0.138477672 | 0.000422298 |  |  |  |  |  |  |  |  |  |
| SEPTIN1 | 0.136581851 | 0.000466224 |  |  |  |  |  |  |  |  |  |
| ENTPD1 | 0.134857883 | 0.002233159 |  |  |  |  |  |  |  |  |  |
| BIRC3 | 0.133901141 | 0.000449544 |  |  |  |  |  |  |  |  |  |
| FAM47E-STBD1 | 0.132246424 | 1.75417E-05 |  |  |  |  |  |  |  |  |  |
| LPXN | 0.130375416 | 0.000370344 |  |  |  |  |  |  |  |  |  |
| HOXB2 | 0.12731206 | 0.001919032 |  |  |  |  |  |  |  |  |  |
| MAP4K1 | 0.12675545 | 0.000576261 |  |  |  |  |  |  |  |  |  |
| PRF1 | 0.126119342 | 0.000181451 |  |  |  |  |  |  |  |  |  |
| FMNL1 | 0.125963736 | 3.87968E-05 |  |  |  |  |  |  |  |  |  |
| COLGALT2 | 0.124063854 | 0.000486054 |  |  |  |  |  |  |  |  |  |
| LST1 | 0.124063002 | 0.000744621 |  |  |  |  |  |  |  |  |  |
| CSF2 | 0.123445064 | 0.000779157 |  |  |  |  |  |  |  |  |  |
| WAS | 0.122665142 | 0.000221106 |  |  |  |  |  |  |  |  |  |
| ETS1 | 0.122155675 | 9.6142E-05 |  |  |  |  |  |  |  |  |  |
| GZMM | 0.121737431 | 0.001531609 |  |  |  |  |  |  |  |  |  |
| BCL11B | 0.12013738 | 0.000532679 |  |  |  |  |  |  |  |  |  |
| LCE1E | 0.120020389 | 0.003109959 |  |  |  |  |  |  |  |  |  |
| GNG2 | 0.117574036 | 0.000167224 |  |  |  |  |  |  |  |  |  |
| CD226 | 0.115045746 | 0.001212194 |  |  |  |  |  |  |  |  |  |
| ICAM3 | 0.114046478 | 0.000368394 |  |  |  |  |  |  |  |  |  |
| RIMKLB | 0.113941589 | 0.000156734 |  |  |  |  |  |  |  |  |  |
| PTGER2 | 0.112118193 | 0.000172046 |  |  |  |  |  |  |  |  |  |
| APOL6 | 0.112076483 | 0.000113594 |  |  |  |  |  |  |  |  |  |
| HPGD | 0.111072373 | 0.001521291 |  |  |  |  |  |  |  |  |  |
| HLA-DPB1 | 0.110554355 | 0.000664197 |  |  |  |  |  |  |  |  |  |
| PCED1B | 0.107920753 | 0.001284131 |  |  |  |  |  |  |  |  |  |
| SLC2A3 | 0.106741223 | 0.00069398 |  |  |  |  |  |  |  |  |  |
| PATL2 | 0.106718304 | 0.000863755 |  |  |  |  |  |  |  |  |  |
| FERMT3 | 0.102828924 | 8.84459E-05 |  |  |  |  |  |  |  |  |  |
| EVL | 0.098020866 | 0.000151656 |  |  |  |  |  |  |  |  |  |
| GPRIN3 | 0.097541026 | 0.001106797 |  |  |  |  |  |  |  |  |  |
| QPRT | 0.097475102 | 0.000215129 |  |  |  |  |  |  |  |  |  |
| AL445685.3 | 0.095943128 | 6.93895E-12 |  |  |  |  |  |  |  |  |  |
| TRAF1 | 0.092528858 | 0.000161726 |  |  |  |  |  |  |  |  |  |
| UBA7 | 0.089937355 | 0.000158131 |  |  |  |  |  |  |  |  |  |
| PLAC8 | 0.088076183 | 0.00053572 |  |  |  |  |  |  |  |  |  |
| LAG3 | 0.086197211 | 2.29565E-05 |  |  |  |  |  |  |  |  |  |
| ZFP91-CNTF | 0.084667553 | 4.89165E-07 |  |  |  |  |  |  |  |  |  |
| LAT | 0.083798056 | 0.000769261 |  |  |  |  |  |  |  |  |  |
| IL10RA | 0.08156647 | 0.000194398 |  |  |  |  |  |  |  |  |  |
| HIC1 | 0.078037934 | 0.002451848 |  |  |  |  |  |  |  |  |  |
| PIK3IP1 | 0.077430838 | 0.000355561 |  |  |  |  |  |  |  |  |  |
| TNFAIP3 | 0.073253671 | 6.4786E-05 |  |  |  |  |  |  |  |  |  |
| SYNGR4 | 0.07323727 | 2.78467E-05 |  |  |  |  |  |  |  |  |  |
| GADD45G | 0.071268707 | 3.30094E-05 |  |  |  |  |  |  |  |  |  |
| CXCR4 | 0.067980449 | 0.000211649 |  |  |  |  |  |  |  |  |  |
| ADTRP | 0.066070447 | 0.001273405 |  |  |  |  |  |  |  |  |  |
| AC008770.2 | 0.06292593 | 6.9156E-05 |  |  |  |  |  |  |  |  |  |
| EIF4EBP3 | 0.062910237 | 0.000230684 |  |  |  |  |  |  |  |  |  |
| NCF4 | 0.058765652 | 5.62253E-05 |  |  |  |  |  |  |  |  |  |
| RASGRP1 | 0.058164569 | 8.17408E-05 |  |  |  |  |  |  |  |  |  |
| TNFSF10 | 0.055611728 | 0.000163053 |  |  |  |  |  |  |  |  |  |
| AOAH | 0.054270459 | 9.76097E-05 |  |  |  |  |  |  |  |  |  |
| NELL2 | 0.053959866 | 0.000375056 |  |  |  |  |  |  |  |  |  |
| AC026954.2 | 0.053865814 | 3.80077E-07 |  |  |  |  |  |  |  |  |  |
| SUMO4 | 0.052547326 | 5.80154E-05 |  |  |  |  |  |  |  |  |  |
| CLEC2D | 0.052425203 | 0.00028779 |  |  |  |  |  |  |  |  |  |
| PSMB8 | 0.051060549 | 0.000204235 |  |  |  |  |  |  |  |  |  |
| DOCK11 | 0.050818443 | 0.000229611 |  |  |  |  |  |  |  |  |  |
| KRTAP3-1 | 0.050343215 | 0.00016268 |  |  |  |  |  |  |  |  |  |
| HCST | 0.047496293 | 0.004479667 |  |  |  |  |  |  |  |  |  |
| ACAP1 | 0.045927048 | 3.8102E-05 |  |  |  |  |  |  |  |  |  |
| VIM | 0.045681633 | 2.36589E-05 |  |  |  |  |  |  |  |  |  |
| GPSM3 | 0.045409296 | 0.000110362 |  |  |  |  |  |  |  |  |  |
| GET1-SH3BGR | 0.044907775 | 1.49324E-06 |  |  |  |  |  |  |  |  |  |
| ITGB2 | 0.04320099 | 7.41476E-05 |  |  |  |  |  |  |  |  |  |
| HLA-DRB5 | 0.043082923 | 0.00666008 |  |  |  |  |  |  |  |  |  |
| SPOCK2 | 0.042832817 | 3.44784E-05 |  |  |  |  |  |  |  |  |  |
| SELPLG | 0.041975779 | 0.000176268 |  |  |  |  |  |  |  |  |  |
| ARHGDIB | 0.040693966 | 3.8118E-05 |  |  |  |  |  |  |  |  |  |
| CD37 | 0.040527063 | 5.76759E-05 |  |  |  |  |  |  |  |  |  |
| GBP2 | 0.038922214 | 5.49238E-05 |  |  |  |  |  |  |  |  |  |
| CTSS | 0.037845928 | 6.92749E-05 |  |  |  |  |  |  |  |  |  |
| PDE4D | 0.034631766 | 2.85341E-05 |  |  |  |  |  |  |  |  |  |
| CEACAM21 | 0.03459105 | 0.000388244 |  |  |  |  |  |  |  |  |  |
| NPHP3-ACAD11 | 0.034586193 | 1.31187E-15 |  |  |  |  |  |  |  |  |  |
| TRIM22 | 0.031442804 | 1.2826E-05 |  |  |  |  |  |  |  |  |  |
| MYO1F | 0.02799512 | 8.60631E-06 |  |  |  |  |  |  |  |  |  |
| TAPBPL | 0.026324384 | 6.59827E-05 |  |  |  |  |  |  |  |  |  |
| ITGB7 | 0.023791223 | 3.53482E-05 |  |  |  |  |  |  |  |  |  |
| CD96 | 0.023064893 | 6.03021E-05 |  |  |  |  |  |  |  |  |  |
| AC116366.2 | 0.019724197 | 1.34623E-28 |  |  |  |  |  |  |  |  |  |
| LBH | 0.018234611 | 1.03511E-06 |  |  |  |  |  |  |  |  |  |
| LCP1 | 0.016221013 | 9.81056E-06 |  |  |  |  |  |  |  |  |  |
| CLEC2B | 0.0145604 | 0.001782131 |  |  |  |  |  |  |  |  |  |
| TNFRSF1B | 0.011736978 | 4.46201E-06 |  |  |  |  |  |  |  |  |  |
| ZNF497 | 0.009397829 | 8.25453E-28 |  |  |  |  |  |  |  |  |  |
| CD52 | 0.009107339 | 0.000138718 |  |  |  |  |  |  |  |  |  |
| LCK | 0.008371313 | 1.81569E-06 |  |  |  |  |  |  |  |  |  |
| GMFG | 0.007773601 | 0.00024547 |  |  |  |  |  |  |  |  |  |
| HCLS1 | 0.007526153 | 9.4512E-06 |  |  |  |  |  |  |  |  |  |
| LSP1 | 0.007516278 | 4.79927E-06 |  |  |  |  |  |  |  |  |  |
| IRF4 | 0.007485051 | 4.09753E-07 |  |  |  |  |  |  |  |  |  |
| AC087721.2 | 0.006846682 | 4.07404E-23 |  |  |  |  |  |  |  |  |  |
| CD74 | 0.006193055 | 9.48056E-06 |  |  |  |  |  |  |  |  |  |
| ICAM2 | 0.004291177 | 7.07028E-10 |  |  |  |  |  |  |  |  |  |
| IL32 | 0.003484692 | 1.11158E-05 |  |  |  |  |  |  |  |  |  |
| LAPTM5 | 0.003193703 | 2.2189E-06 |  |  |  |  |  |  |  |  |  |
| SRGN | 0.003167938 | 9.03025E-06 |  |  |  |  |  |  |  |  |  |
| GNLY | 0.001046435 | 1.36151E-06 |  |  |  |  |  |  |  |  |  |
|  |  |  |  |  |  |  |  |  |  |  |  |
|  |  |  |  |  |  |  |  |  |  |  |  |
|  |  |  |  |  |  |  |  |  |  |  |  |
|  |  |  |  |  |  |  |  |  |  |  |  |
|  |  |  |  |  |  |  |  |  |  |  |  |
|  |  |  |  |  |  |  |  |  |  |  |  |
|  |  |  |  |  |  |  |  |  |  |  |  |
|  |  |  |  |  |  |  |  |  |  |  |  |
|  |  |  |  |  |  |  |  |  |  |  |  |
|  |  |  |  |  |  |  |  |  |  |  |  |
|  |  |  |  |  |  |  |  |  |  |  |  |
|  |  |  |  |  |  |  |  |  |  |  |  |
|  |  |  |  |  |  |  |  |  |  |  |  |
|  |  |  |  |  |  |  |  |  |  |  |  |
|  |  |  |  |  |  |  |  |  |  |  |  |
|  |  |  |  |  |  |  |  |  |  |  |  |
|  |  |  |  |  |  |  |  |  |  |  |  |
|  |  |  |  |  |  |  |  |  |  |  |  |
|  |  |  |  |  |  |  |  |  |  |  |  |
|  |  |  |  |  |  |  |  |  |  |  |  |
|  |  |  |  |  |  |  |  |  |  |  |  |
|  |  |  |  |  |  |  |  |  |  |  |  |
|  |  |  |  |  |  |  |  |  |  |  |  |
|  |  |  |  |  |  |  |  |  |  |  |  |
|  |  |  |  |  |  |  |  |  |  |  |  |
|  |  |  |  |  |  |  |  |  |  |  |  |
|  |  |  |  |  |  |  |  |  |  |  |  |
|  |  |  |  |  |  |  |  |  |  |  |  |
|  |  |  |  |  |  |  |  |  |  |  |  |
|  |  |  |  |  |  |  |  |  |  |  |  |
|  |  |  |  |  |  |  |  |  |  |  |  |
|  |  |  |  |  |  |  |  |  |  |  |  |
|  |  |  |  |  |  |  |  |  |  |  |  |
|  |  |  |  |  |  |  |  |  |  |  |  |
|  |  |  |  |  |  |  |  |  |  |  |  |
|  |  |  |  |  |  |  |  |  |  |  |  |
|  |  |  |  |  |  |  |  |  |  |  |  |
|  |  |  |  |  |  |  |  |  |  |  |  |
|  |  |  |  |  |  |  |  |  |  |  |  |
|  |  |  |  |  |  |  |  |  |  |  |  |
|  |  |  |  |  |  |  |  |  |  |  |  |
|  |  |  |  |  |  |  |  |  |  |  |  |
|  |  |  |  |  |  |  |  |  |  |  |  |
|  |  |  |  |  |  |  |  |  |  |  |  |
|  |  |  |  |  |  |  |  |  |  |  |  |
|  |  |  |  |  |  |  |  |  |  |  |  |
|  |  |  |  |  |  |  |  |  |  |  |  |
|  |  |  |  |  |  |  |  |  |  |  |  |
|  |  |  |  |  |  |  |  |  |  |  |  |
|  |  |  |  |  |  |  |  |  |  |  |  |
|  |  |  |  |  |  |  |  |  |  |  |  |
|  |  |  |  |  |  |  |  |  |  |  |  |
|  |  |  |  |  |  |  |  |  |  |  |  |
|  |  |  |  |  |  |  |  |  |  |  |  |
|  |  |  |  |  |  |  |  |  |  |  |  |
|  |  |  |  |  |  |  |  |  |  |  |  |
|  |  |  |  |  |  |  |  |  |  |  |  |
|  |  |  |  |  |  |  |  |  |  |  |  |
|  |  |  |  |  |  |  |  |  |  |  |  |
|  |  |  |  |  |  |  |  |  |  |  |  |
|  |  |  |  |  |  |  |  |  |  |  |  |
|  |  |  |  |  |  |  |  |  |  |  |  |
|  |  |  |  |  |  |  |  |  |  |  |  |
|  |  |  |  |  |  |  |  |  |  |  |  |
|  |  |  |  |  |  |  |  |  |  |  |  |
|  |  |  |  |  |  |  |  |  |  |  |  |
|  |  |  |  |  |  |  |  |  |  |  |  |
|  |  |  |  |  |  |  |  |  |  |  |  |
|  |  |  |  |  |  |  |  |  |  |  |  |
|  |  |  |  |  |  |  |  |  |  |  |  |
|  |  |  |  |  |  |  |  |  |  |  |  |
|  |  |  |  |  |  |  |  |  |  |  |  |
|  |  |  |  |  |  |  |  |  |  |  |  |
|  |  |  |  |  |  |  |  |  |  |  |  |
|  |  |  |  |  |  |  |  |  |  |  |  |
|  |  |  |  |  |  |  |  |  |  |  |  |
|  |  |  |  |  |  |  |  |  |  |  |  |
|  |  |  |  |  |  |  |  |  |  |  |  |
|  |  |  |  |  |  |  |  |  |  |  |  |
|  |  |  |  |  |  |  |  |  |  |  |  |
|  |  |  |  |  |  |  |  |  |  |  |  |
|  |  |  |  |  |  |  |  |  |  |  |  |
|  |  |  |  |  |  |  |  |  |  |  |  |
|  |  |  |  |  |  |  |  |  |  |  |  |
|  |  |  |  |  |  |  |  |  |  |  |  |
|  |  |  |  |  |  |  |  |  |  |  |  |
|  |  |  |  |  |  |  |  |  |  |  |  |
|  |  |  |  |  |  |  |  |  |  |  |  |
|  |  |  |  |  |  |  |  |  |  |  |  |
|  |  |  |  |  |  |  |  |  |  |  |  |
|  |  |  |  |  |  |  |  |  |  |  |  |
|  |  |  |  |  |  |  |  |  |  |  |  |
|  |  |  |  |  |  |  |  |  |  |  |  |
|  |  |  |  |  |  |  |  |  |  |  |  |
|  |  |  |  |  |  |  |  |  |  |  |  |
|  |  |  |  |  |  |  |  |  |  |  |  |
|  |  |  |  |  |  |  |  |  |  |  |  |
|  |  |  |  |  |  |  |  |  |  |  |  |
